# Effect of Vacuum Heat Treatment on the Structural-Phase State of Detonation-Sprayed NiCrAl Coatings

**DOI:** 10.3390/ma19184023

**Published:** 2026-09-21

**Authors:** Maulet Meruyert, Zhuldyz Sagdoldina, Nurkhat Bimakhan

**Affiliations:** Research Center «Surface Engineering and Tribology», Sarsen Amanzholov East Kazakhstan University, 070002 Ust-Kamenogorsk, Kazakhstan; mauletmeruert@gmail.com (M.M.); nurhat130820@gmail.com (N.B.)

**Keywords:** NiCrAl coatings, detonation spraying, vacuum heat treatment, microstructure, XRD, phase composition, β-NiAl, interdiffusion, oxide phases, roughness

## Abstract

The effect of vacuum heat treatment on the structural-phase state of detonation-sprayed NiCrAl coatings was investigated. The samples were annealed in vacuum at temperatures of 800, 900, 1000, and 1100 °C with a holding time of 4 h. It was established that the as-sprayed coating is characterized predominantly by the presence of β-NiAl and a non-equilibrium lamellar structure formed under conditions of high-velocity detonation deposition and rapid particle cooling. At 800 °C, diffusion processes are activated, and a more complex multiphase state involving Ni–Al- and Cr-containing intermetallic phases is formed. Increasing the temperature to 900 °C promotes further structural and chemical homogenization of the coating while retaining β-NiAl as one of the main phases. At 1000 °C, along with continued homogenization, Cr_2_O_3_ and NiO appear, while interdiffusion between the coating and the Fe-containing substrate becomes more pronounced near the interface. After treatment at 1100 °C, the most profound phase transformation is observed, with formation of Al_2_O_3_, Cr_2_O_3_, NiO, and NiAl_2_O_4_, significant redistribution of the Ni–Al intermetallic constituent, and formation of a chemically heterogeneous transition zone with local microvoids. Increasing temperature is also accompanied by changes in surface morphology and a moderate decrease in roughness. Surface roughness showed a non-monotonic temperature dependence, with Ra values of 6, 5.3, 5.3, and 5.9 μm after heat treatment at 800, 900, 1000, and 1100 °C, respectively; the minimum roughness was obtained at 900 °C.

## 1. Introduction

Ni–Cr–Al-containing coatings are among the most widely used metallic systems for the surface protection of heat-resistant alloys and steels operating under conditions of high-temperature oxidation, hot corrosion, and thermal cyclic loading. NiCrAl and NiCrAlY coatings are most widely used as standalone heat-resistant layers and metallic bond coats in thermal barrier coating systems for gas turbine and aircraft engine components [1,2,3,4]. The protective effect of such materials is primarily attributed to the ability of Al and Cr to participate in the formation of relatively stable Al_2_O_3_ and Cr_2_O_3_ oxide layers. At the same time, the service life depends not only on the overall Al and Cr contents but also on their local distribution, the phase state of the matrix, the depletion kinetics of the β-NiAl-like phase, the composition of the thermally grown oxide layer, and the defect state of the as-sprayed coating [5,6,7,8]. Studies on MCrAlY coatings show that the microstructure of the as-sprayed coating significantly affects the early stages of oxidation, while internal oxide layers can accelerate local depletion of the β-phase [3,4,5].

For Ni–Cr–Al systems, the balance between the Al content and the mechanical stability of the coating is of particular importance. A sufficient Al reserve is necessary to maintain the protective alumina film; however, the phase state of Al-rich regions and the development of intermetallic constituents can significantly affect the ductility and cracking susceptibility of the coating. Under prolonged high-temperature exposure, Al consumption for oxide formation and its interdiffusion with the substrate lead to the evolution of β-NiAl-containing regions and changes in the relative proportions of the γ, β, and γ′ constituents [3,4,5,6,7]. For detonation-sprayed Ni–Cr–Al coatings subjected to cyclic oxidation at 1000 °C, Al_2_O_3_ and Cr_2_O_3_ were observed along with secondary NiO and spinel oxides; investigation of the gradient structure showed more pronounced Al_2_O_3_ peaks and retention of a higher Cr content in certain regions of the coating [1].

One promising method for depositing NiCrAl coatings is detonation thermal spraying. Unlike continuous flame-spraying processes, the acceleration and heating of powder particles during detonation spraying occur impulsively under the action of detonation products. The high kinetic energy of the particles ensures their intense deformation upon impact with the substrate, promotes the formation of dense contact between individual splats, and enables the production of coatings with high adhesion strength [9,10,11,12]. Computer-controlled detonation systems additionally allow variation in the barrel filling with the fuel–oxygen mixture, powder dosing, and cycle sequence, thereby controlling the thermal and velocity state of the particles [12]. Studies on Ni–Cr and Ni–Cr–Al have directly shown that the detonation barrel filling parameters have a noticeable effect on the chemical and phase composition of the coating; in particular, changes in the spraying conditions can affect Al retention and the final phase distribution [9,13]. However, the high density of detonation-sprayed coatings does not imply the complete absence of structural defects. Like other thermal-sprayed coatings, they are formed by the successive deposition of deformed particles and therefore may contain interlamellar boundaries, local discontinuities, pores, microcracks, insufficiently melted or non-uniformly deformed particles, and oxide inclusions [14,15].

Another fundamental feature of sprayed coatings is the presence of residual stresses. Their final state is determined by the competition between impact-induced surface hardening by high-velocity particles, shrinkage stresses during rapid cooling of splats, differences in the coefficients of thermal expansion between the coating and the substrate, and non-uniformity of the temperature field. Depending on the combination of these factors, local stresses can contribute both to coating densification and to the formation of interlamellar microcracks or weakening of the interface. Therefore, the high initial adhesion of a detonation-sprayed coating does not eliminate the need for stabilization treatment, especially when the component is intended for long-term high-temperature service.

Studies on heat-treated MCrAlY coatings show that enhanced interfacial diffusion and the development of metallurgical bonding at the substrate interface can significantly alter the adhesion mechanism compared with the as-sprayed state [16,17,18]. For this reason, a wide range of post-treatment methods has been developed for thermal-sprayed coatings. Thermal annealing in air or another oxidizing atmosphere activates diffusion, recrystallization, and relaxation processes; however, it simultaneously causes the growth of an oxide film and oxygen penetration along open pores and interlamellar boundaries [3,4,5,6,7,8]. Laser and electron-beam remelting make it possible to locally eliminate a significant portion of the lamellar boundaries and obtain a more homogeneous remelted surface layer; such approaches have been investigated for Ni–20Cr and CoNiCrAlY [19,20,21]. However, this approach primarily modifies the near-surface region and is not fully equivalent to bulk annealing of the entire coating together with the interface. Hot isostatic pressing (HIP) combines elevated temperature with isostatic pressure and can promote intensive closure of internal pores and the development of interparticle bonding; its effectiveness for thermal-sprayed systems has been demonstrated in the studies by Khor and Loh et al. [22]. At the same time, HIP is technologically more complex than conventional vacuum annealing and is not always justified for large coated components. Spark plasma sintering also demonstrates the possibility of substantial restructuring of the sprayed structure, although published studies in this area are predominantly related to ceramic coatings [23].

Against this background, vacuum heat treatment (VHT) occupies an intermediate position: it provides bulk thermal exposure of the coating and the interface without the application of external isostatic pressure and at a significantly lower oxygen potential than annealing in air. For systems similar to NiCrAl, the published treatment conditions vary considerably. Li et al. treated APS-Ni22Cr10Al1Y at 900 and 1100 °C for 1 h in a dynamic vacuum of approximately 10^−1^ Pa [2]. Ghadami et al. investigated nanostructured HVOF-NiCoCrAlY at 1100 °C with holding times of 1, 2, and 5 h at a pressure of approximately 10^−3^ Pa [24]. For HVOF-NiCrAlY coatings, the effect of preliminary VHT in the temperature range of 1000–1200 °C has been investigated [25], whereas Keller et al. showed that not only the temperature but also the specific residual pressure and gas atmosphere—from high vacuum on the order of 10^−3^–10^−2^ Pa to conditions of approximately 10^−1^ Pa in Ar—can alter the chemical state of the MCrAlY surface [26]. Therefore, the term “vacuum heat treatment” itself is insufficient for interpreting the results: pressure, temperature, holding time, heating rate, and cooling rate should be considered as interrelated processing variables.

Despite the significant number of publications on the heat treatment of MCrAlY coatings, most systematic studies have been carried out for APS, HVOF, and VPS coatings [2,16,17,18,26]. For detonation-sprayed Ni–Cr–Al coatings themselves, the studies identified are devoted mainly to controlling the deposition conditions and coating gradient structure [9,13], high-temperature oxidation [1], or the investigation of technologically similar but compositionally different materials [10,14,15]. Therefore, the question of how the specific initial structure formed by high-velocity detonation deposition of NiCrAl transforms specifically during subsequent vacuum treatment remains insufficiently studied.

Although hardness and residual stresses can influence the mechanical stability of thermally sprayed coatings, the present study does not include direct residual-stress or microhardness measurements. Mechanical performance is therefore discussed only in relation to the experimentally determined critical scratch load, while the main focus remains on the temperature-dependent phase, microstructural, and interfacial evolution of the NiCrAl coating.

Thus, the aim of this study is to systematically determine how vacuum heat treatment in the temperature range of 800–1100 °C affects the structural and phase evolution of detonation-sprayed NiCrAl coatings deposited on 14MoV6-3 steel. The specific objectives were: (i) to identify temperature-dependent changes in the phase composition of the coating by XRD; (ii) to evaluate the development and extent of coating–substrate interdiffusion using cross-sectional SEM/EDS analysis; (iii) to determine the evolution of surface morphology and roughness after heat treatment; and (iv) to establish the temperature range at which oxide phases become detectable under the applied vacuum-treatment conditions. Particular attention was paid to distinguishing the temperature range in which structural homogenization predominates from that in which intensive interdiffusion and oxidation become significant. This information is relevant to the selection of post-treatment conditions for NiCrAl protective coatings intended for high-temperature components, where excessive interdiffusion and oxide formation may compromise coating stability. The study therefore addresses the lack of systematic data on the vacuum-induced structural evolution of detonation-sprayed NiCrAl coatings, which has been much less investigated than APS-, HVOF-, and VPS-deposited MCrAlY systems.

## 2. Materials and Methods

### 2.1. Substrate Material and Surface Preparation

Low-alloy 14MoV6-3 steel, conforming to ISO 4955 [27], was used as the substrate material. Supplier-specific information was not available for the material batch used in this study. Since 14MoV6-3 is a commercial low-alloy steel rather than a high-purity material, the substrate is identified by its standardized grade designation rather than by a nominal purity value. Square specimens measuring 15 mm × 15 mm with a thickness of 3 mm were prepared from the steel. The nominal chemical composition and representative mechanical properties of 14MoV6-3 steel are summarized in Table 1. Since a batch-specific material certificate was unavailable, the values presented in the table correspond to the standardized composition and typical properties reported for this steel grade and should not be interpreted as independently measured values for the particular substrate batch used in this study.

14MoV6-3 steel was selected as the substrate because it is a Cr–Mo–V low-alloy steel developed for elevated-temperature pressure applications and possesses a combination of high-temperature strength, creep resistance, and sufficient ductility. These characteristics make it a technically relevant substrate for evaluating NiCrAl coatings intended for thermal protection of steel components. In addition, the Fe-based substrate provides a suitable model system for examining temperature-dependent interdiffusion between the Ni–Cr–Al coating and a ferritic low-alloy steel during vacuum heat treatment.

Before coating deposition, the substrate surfaces were subjected to sequential mechanical preparation. Grinding and polishing were performed using a METAPOL 2000P grinding-polishing machine (Laizhou Lyric Testing Equipment Co., Ltd., Laizhou, China). The specimens were sequentially ground using SiC abrasive papers from P120 to P2000 grit. Each subsequent abrasive grade was applied after the scratches produced during the preceding grinding step had been removed; therefore, the duration of each grinding stage was controlled by the surface condition rather than by a fixed processing time. After grinding, the specimens were subjected to abrasive blasting using quartz sand as the blasting medium. The blasting chamber had a nominal productivity of 1–3 m^2^/h, an adjustable abrasive-jet angle of 15–90°, and an operating pressure range of 3.5–7 atm. Abrasive blasting was performed until a visually uniform roughened surface was obtained, providing increased surface roughness and improved mechanical interlocking for subsequent detonation spraying.

The completion of each grinding step was assessed by visual inspection of the specimen surface. Grinding with a given abrasive grade was continued until scratches and surface features produced during the preceding preparation step were no longer visible. After the final grinding stage, abrasive blasting with quartz sand was applied until a visually uniform matte surface was obtained over the entire area intended for coating deposition. This procedure was used to remove residual surface films and machining-related irregularities and to obtain a reproducible surface condition prior to detonation spraying. No separate surface-chemical analysis was performed after mechanical preparation; therefore, the effectiveness of the preparation was assessed operationally from the uniformity of the prepared surface rather than by direct quantitative measurement of residual contamination or oxide-film thickness.

### 2.2. NiCrAl Feedstock Powder and Its Characterization

**Figure 1:**
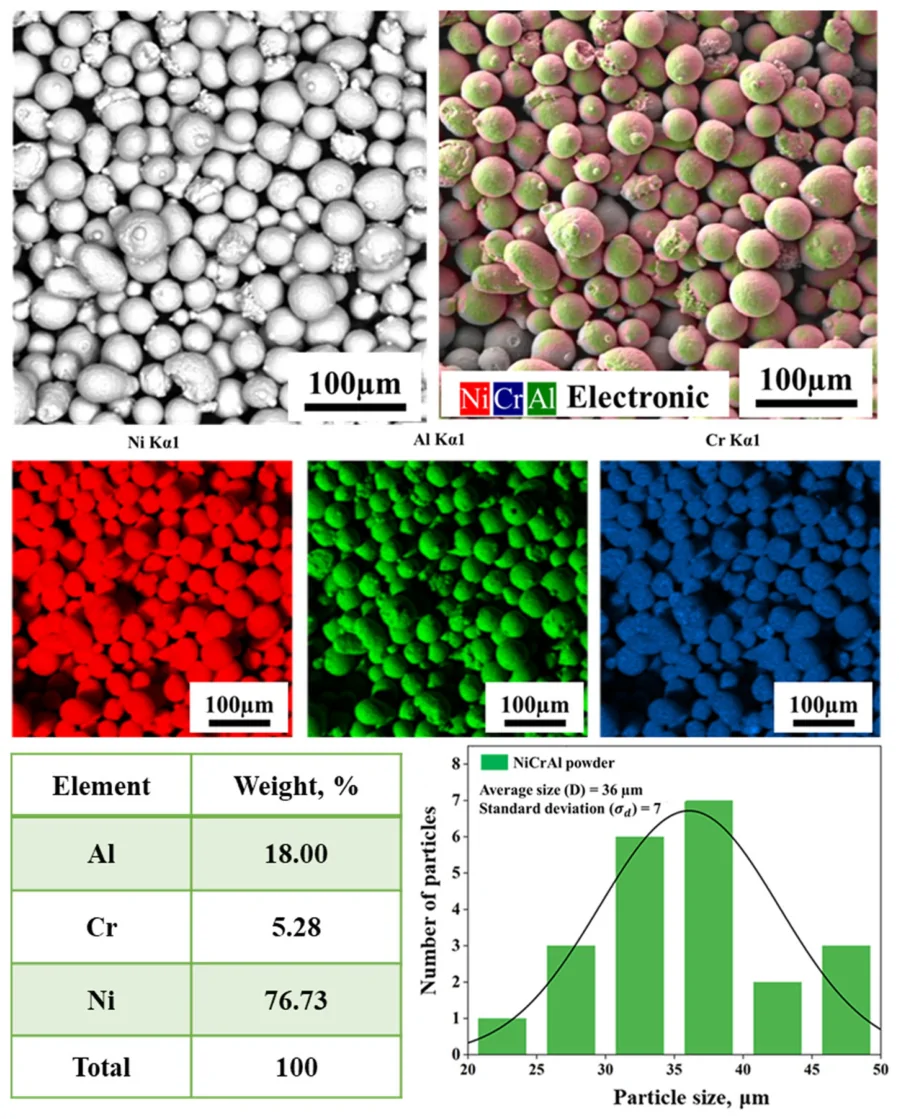
Morphology, particle-size distribution, and elemental distribution of the initial NiCrAl powder. In the elemental maps, Ni, Al, and Cr are represented in red, green, and blue, respectively. The black curve represents the fitted normal distribution of particle sizes.

NiCrAl alloy powder (Qinghe County Tebo Metal Materials Co., Ltd., Xingtai, China) was used as the feedstock material for detonation spraying. According to the supplier, the nominal particle-size range was 15–45 μm. The particle morphology, size characteristics, and distribution of the main elements were investigated using scanning electron microscopy (CIQTEK, Hefei, China) and energy-dispersive microanalysis (Figure 1). According to the SEM data, the powder consisted predominantly of spherical and near-spherical particles. Along with individual particles, small agglomerates and particles with local surface irregularities were observed. The particle-size distribution was determined from SEM images by digital image analysis using ImageJ software (version 1.54). SEM-based image analysis showed an experimentally observed particle-size range of approximately 20–50 μm, with an average particle size of 36 ± 7 μm. The exact number of individual particles included in the original ImageJ analysis was not retained in the experimental record; therefore, the particle-size distribution is reported as 36 ± 7 μm (mean ± standard deviation), while no unsupported value of n is introduced. The relatively narrow particle-size distribution and predominantly spherical morphology contribute to uniform powder feeding and more stable interaction of the particles with the detonation products. EDS analysis of the initial NiCrAl powder confirmed Ni, Al, and Cr as the principal metallic constituents. The representative composition was approximately 76.73 wt.% Ni, 18.00 wt.% Al, and 5.28 wt.% Cr. The exact number of individual EDS measurements used to obtain these values was not retained in the experimental record; therefore, the reported composition is presented as representative rather than as a statistically averaged value. The elemental distribution maps show the presence of Ni, Al, and Cr throughout the investigated powder particles without pronounced large-scale segregation. This relatively uniform elemental distribution provides favorable conditions for the formation of a compositionally homogeneous Ni–Cr–Al coating during subsequent detonation spraying.

Compared with conventional NiCrAlY feedstock compositions, the present powder contains a substantially higher Al content and a lower Cr content and does not contain Y. For example, a NiCrAlY powder with a nominal composition of Ni–20Cr–10Al–0.5Y (wt.%) has been reported for detonation-sprayed NiCrAlY coatings [28]. The higher Al content of the present NiCrAl powder is expected to favor a larger contribution of Al-rich β-NiAl and related Ni–Al intermetallic phases, whose stability and redistribution strongly depend on temperature and alloy composition [29]. In conventional MCrAlY systems, β-NiAl acts as an important Al reservoir during high-temperature exposure, whereas Y additions can improve the adherence and stability of alumina-containing oxide scales through the reactive-element effect [30]. Since the present powder does not contain Y, this reactive-element contribution is absent. Therefore, the differences in Al, Cr, and Y contents should be considered when comparing the phase evolution of the present NiCrAl coating with conventional NiCrAlY systems.

### 2.3. Detonation Spraying Procedure

The NiCrAl coatings were deposited using a CCDS2000 computer-controlled detonation spraying system (LIH SB RAS, Novosibirsk, Russia), which provides programmable control of the operating cycle and stable reproduction of the specified process parameters [9,31]. The use of an automated control system made it possible to maintain constant gas mixture composition, barrel filling ratio, operating cycle frequency, powder feed rate, and other parameters determining the energy state of the particles before their interaction with the substrate. Oxygen and acetylene (O_2_–C_2_H_2_) were used as the fuel gas mixture. Detonation of this mixture generates a short-duration, high-temperature, high-velocity gas flow that heats and accelerates the NiCrAl particles within the barrel of the system. After exiting the barrel, the particles reach the substrate surface with high kinetic energy, undergo intense deformation upon impact, and successively form a dense coating layer. The O_2_/C_2_H_2_ ratio is one of the main parameters of the detonation process, as it determines the temperature of the reaction products, the gas flow velocity, and the nature of the thermal effect on the powder particles. Changes in the composition of the detonating mixture can affect the degree of particle heating, their plasticity at the moment of impact with the substrate, the intensity of oxidation processes, and the likelihood of phase transformations occurring directly during spraying. For the multicomponent Ni–Cr–Al system, this is particularly important because Ni, Cr, and Al differ in their chemical activity and tendency to interact with oxygen. The spraying conditions were selected to ensure stable particle acceleration and the formation of a continuous coating while limiting excessive overheating and oxidation of the powder material. The sequential deposition of individual powder portions under repeated detonation pulses resulted in the formation of a layered structure characteristic of detonation-sprayed coatings, consisting of deformed and mutually overlapping particles. The main technological parameters used for producing the NiCrAl coatings are presented in Table 2.

The CCDS2000 system was equipped with a straight cylindrical barrel with an internal diameter of 20 mm and an approximate length of 800 mm. NiCrAl powder was introduced into the barrel using nitrogen as the carrier gas. The oxygen-to-acetylene ratio was set to O_2_/C_2_H_2_ = 1.856. The O_2_/C_2_H_2_ value of 1.856 denotes the preset oxygen-to-acetylene ratio used for the formation of the explosive gas mixture in the CCDS2000 system. The barrel filling fraction was 50%, the spraying distance was 150 mm, and 20 detonation shots were used to produce the coating. Under these conditions, the as-sprayed coating thickness was approximately 85–90 μm, as determined from cross-sectional SEM observations. The exact powder feed rate and total spraying time were not retained in the experimental records and therefore cannot be reported reliably.

The values listed in Table 2 correspond to the nominal process settings used during deposition. Instrument-specific uncertainties or control tolerances were not retained in the experimental records; therefore, unsupported uncertainty values have not been introduced. Where applicable, experimentally observed ranges are reported, for example, the as-sprayed coating thickness of approximately 85–90 μm.

To ensure the comparability of the vacuum heat treatment results, the NiCrAl coating was deposited on all exposed surfaces of the samples, including the front, side, and rear surfaces. This approach was chosen to eliminate direct contact of the uncoated steel substrate with the residual atmosphere of the vacuum chamber and to minimize the influence of local oxidation or decarburization of exposed steel regions on the results of subsequent analysis. With one-sided NiCrAl deposition, the opposite uncoated surface could participate in additional oxidation–reduction processes and could also create different thermal and chemical effects on the sample. Applying the coating over the entire perimeter reduced the influence of this factor and made it possible to consider the structural changes predominantly as a result of vacuum heat treatment of the NiCrAl/14MoV6-3 system itself.

### 2.4. Vacuum Heat Treatment

**Figure 2:**
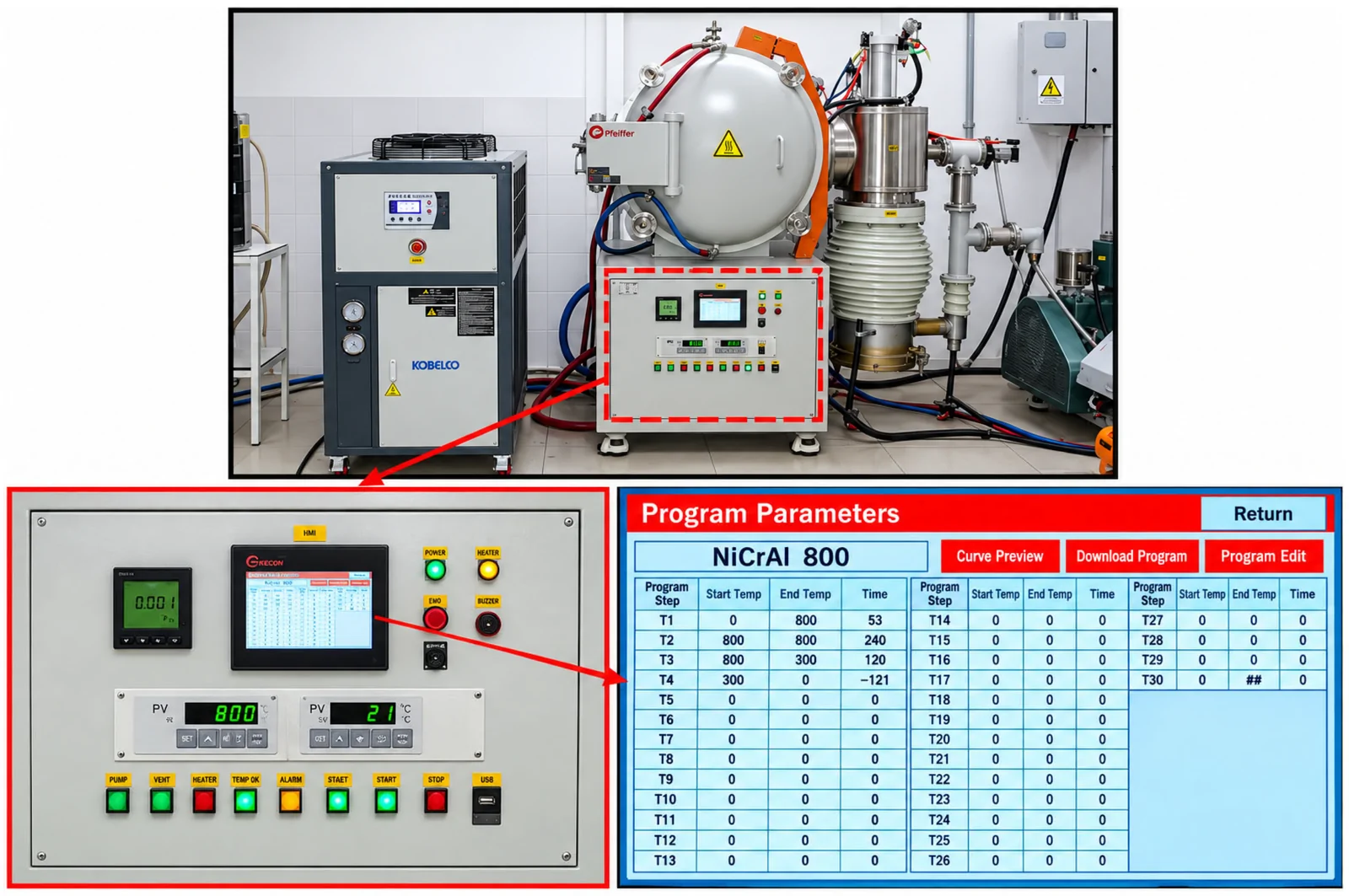
BR-17HVF-8 high-temperature vacuum furnace and programmable control panel used for vacuum heat treatment of detonation-sprayed NiCrAl coatings.

After detonation spraying, the NiCrAl-coated samples were subjected to vacuum heat treatment in a BR-17HVF-8 high-temperature vacuum furnace (Zhengzhou Brother Furnace Co., Ltd., Zhengzhou, China). The furnace has a rated power of 7 kW, a maximum temperature of 1700 °C, a working temperature range of 0–1600 °C, and a chamber size of 200 mm × 200 mm × 200 mm (Figure 2). The furnace is equipped with MoSi_2_-based heating elements and a programmable temperature-control system, which enables the temperature–time treatment cycle to be set and reproduced.

To investigate the effect of temperature on the structural-phase state of the coating, four treatment temperatures were selected: 800, 900, 1000, and 1100 °C. The duration of isothermal holding at each temperature was 4 h. The samples were placed in the working chamber of the furnace so that their surfaces did not come into contact with each other. After sealing the chamber, it was evacuated, and the samples were subsequently heated to the specified temperature. Vacuum was maintained in the furnace chamber throughout the heat-treatment cycle. However, the absolute chamber pressure was not logged during these experiments, and the composition of the residual gas atmosphere was not analyzed. Therefore, the total chamber pressure, oxygen partial pressure, and concentrations of other residual gas species cannot be quantified retrospectively.

The isothermal holding time was counted from the moment the set temperature was reached. After completion of the 4 h isothermal holding period, the samples were cooled directly in the working chamber of the vacuum furnace. The heating/cooling rates were 15.1/4.2, 15.0/3.75, 14.9/3.9, and 14.8/4.0 °C/min for the 800, 900, 1000, and 1100 °C conditions, respectively. The controlled heating and cooling conditions were used to limit sharp temperature gradients and associated thermal stresses in the NiCrAl coating–steel substrate system and to provide reproducible conditions for phase and diffusion-induced structural evolution. The technological parameters of the vacuum heat treatment of the detonation-sprayed NiCrAl coatings are summarized in Table 3.

The vacuum heat treatment temperatures of 800, 900, 1000, and 1100 °C were selected for the sequential investigation of different stages of the structural and phase evolution of the NiCrAl coating. Such a temperature range is in good agreement with literature data on NiCrAlY/MCrAlY systems [32,33,34,35,36,37]. Thus, vacuum treatment of NiCrAlY at 800, 900, and 1000 °C already leads to the formation and gradual development of an interdiffusion zone, with the depth of coating–substrate interaction increasing as the temperature rises [38]. For NiCrAlY coatings after vacuum annealing at 900 °C, changes in the phase proportions of γ-Ni, β-NiAl, γ′-Ni_3_Al, and α-Cr have been experimentally observed, confirming the active development of phase redistribution already within this temperature range [32]. The temperature of 1000 °C is of particular interest because pronounced microstructural changes, the formation of an interdiffusion zone, and the appearance of γ′-Ni_3_Al and α-Cr have previously been observed in NiCrAlY after vacuum holding for 4 h [33]. With a further increase in temperature to 1100 °C, the restructuring of the coating becomes even more intensive: in HVOF-NiCrAlY coatings after vacuum treatment at 1000, 1100, and 1200 °C, changes in the morphology of β-NiAl and γ′-Ni_3_Al were observed, with γ′-Ni_3_Al acquiring a pronounced lamellar structure at 1100 °C, while the coating porosity gradually decreased [25]. In addition, studies of the high-temperature stability of NiCrAlY show that in the 1000–1100 °C range, the relative proportions of the β, γ, and γ′ phases change significantly, diffusion processes intensify, and conditions are created for the formation of stable oxide phases [25,34]. Therefore, the temperature range of 800–1100 °C with a 100 °C interval was selected not arbitrarily, but to cover the sequential transition from initial diffusion relaxation and phase ordering at 800 °C through active homogenization at 900 °C to intensive interdiffusion and profound phase transformation at 1000–1100 °C.

The isothermal holding time of 4 h was selected to provide sufficient time for the development of diffusion-controlled processes in the NiCrAl coating while simultaneously limiting excessive high-temperature degradation of the coating–substrate system. A similar holding time is widely used in vacuum heat treatment of NiCrAlY and NiCoCrAlY coatings. Thus, in a study of a NiCrAlY coating deposited on a Ni-based single-crystal superalloy, vacuum treatment at 1000 °C for 4 h led to pronounced changes in the microstructure and phase composition, the formation of an interdiffusion zone, and the appearance of new phases due to coating–substrate interaction [33]. In addition, vacuum treatment of NiCoCrAlY coatings at 950, 1000, and 1050 °C for 4 h was investigated, and subsequent SEM/EDS and XRD analyses showed pronounced structural changes, improved coating–substrate bonding, and a dependence of the properties on the annealing temperature [35]. Thus, a holding time of 4 h is sufficient for the development of interlamellar, grain-boundary, and interfacial diffusion and makes it possible to reveal temperature-dependent changes in the phase composition without proceeding to excessively prolonged aging. Using the same four-hour holding time at 800, 900, 1000, and 1100 °C also ensures a valid comparison of the investigated conditions, since the treatment duration remains constant while temperature is the primary variable. At the same time, the literature data show that even shorter vacuum treatment of NiCrAlY for 2 h at 1000–1200 °C already leads to substantial transformation of β-NiAl and γ′-Ni_3_Al and a reduction in porosity [25], whereas studies with holding times of 1, 2, and 5 h at 1100 °C demonstrate a pronounced dependence of the structure and oxidation resistance of MCrAlY coatings on the annealing duration [36]. On this basis, a holding time of 4 h was selected as an intermediate duration sufficient to achieve pronounced structural and phase transformation of the coating without excessively prolonged high-temperature exposure.

### 2.5. X-Ray Diffraction Analysis

The phase composition and crystal structure characteristics of the NiCrAl coatings were investigated by X-ray diffraction using a DX-27mini benchtop diffractometer (Dandong Haoyuan Instrument Co., Ltd., Dandong, China). Measurements were performed using Cu-Kα radiation with a wavelength of λ = 1.541 Å. The X-ray tube with a copper anode was operated at a voltage of 30 kV and a current of 10 mA. The diffraction data were analyzed over the 2θ range of 20–90° with a step size of 0.02° and a counting time of 0.5 s per step. The crystalline phases were identified by comparing the experimental diffraction peak positions and relative intensities with reference diffraction patterns using HighScore software (Version:3.0e(3.0.5)) and the PDF-2 powder diffraction database. Phase assignments were based on the agreement of multiple characteristic reflections where possible. The reference diffraction card numbers used for phase identification are summarized in Table 4.

In addition to qualitative phase identification, a semi-quantitative comparison of the phase evolution was performed by integrating the areas of selected characteristic reflections after local baseline subtraction. Because several reflections overlap and the investigated coatings may exhibit preferred orientation and microstructural broadening, this procedure was not used to calculate absolute phase fractions in wt.%. Instead, the integrated areas were normalized to the total area of the selected reflections and are reported as normalized relative diffraction contributions. For β-NiAl, reflections near 31.1°, 44.6°, 65.0°, and 82.3° were considered; for Al_4_Ni_3_, the reflection near 29.3°; for AlNi_3_, the reflection near 25.9°; for Al_3_Ni_2_, the reflection near 51.0°; and for oxide/spinel constituents, combined reflections near 37.0°, 43.2°, and 57.4°. These values are intended only for comparative evaluation of temperature-dependent phase evolution and should not be interpreted as rigorous quantitative phase fractions.

### 2.6. SEM/EDS Characterization and Image Analysis

The microstructure of the coatings was examined by scanning electron microscopy using an SEM3200 scanning electron microscope (CIQTEK Co., Ltd., Hefei, China). Energy-dispersive X-ray spectroscopy (EDS) was performed using an XFlash Detector 730M-300 (Bruker Nano GmbH, Berlin, Germany) coupled to the SEM. The SEM/EDS analysis was carried out on polished cross-sections of the coatings to evaluate the coating morphology, coating–substrate interface, local defects, and elemental distribution. For local compositional characterization of the coatings, EDS point analyses were performed at three representative locations on the investigated coating specimen. One spectrum was acquired at each selected location to assess local compositional variations. In addition, EDS elemental mapping and line-scan analyses were performed where appropriate to evaluate the elemental distribution within the coating and across the coating–substrate interface. For each heat-treatment condition, one representative cross-section was examined by SEM/EDS. One EDS line scan was acquired across the coating–substrate interface for each characterized specimen to evaluate the redistribution of the principal elements through the interfacial region. Therefore, the cross-sectional SEM/EDS and line-scan results are interpreted as representative microstructural and compositional observations rather than as statistically averaged data. Point analysis, elemental mapping, and line scanning were performed for the main elements of the system, including Ni, Al, Cr, Fe, and O. For a semi-quantitative assessment of void-like defects, cross-sectional BSE images were analyzed using ImageJ. The analyzed region was restricted to the coating cross-section, excluding the mounting medium, substrate, scale bars, and annotations. Dark regions corresponding to void-like features were segmented using the same relative grayscale criterion for all conditions, and their area fraction was calculated relative to the analyzed coating area. Because BSE contrast may also arise from local compositional variations, particularly in Al- and O-rich regions, the resulting values are reported as image-based apparent void-area fractions rather than absolute porosity. Linear concentration profiles across the cross-section were used to evaluate the nature of the elemental transition in the interface region and to identify changes associated with interdiffusion after vacuum heat treatment. The combined use of XRD, SEM, and EDS made it possible to correlate phase transformations with changes in the morphology and chemical heterogeneity of the coating at different annealing temperatures. This comprehensive approach was used to analyze the evolution of Ni–Al- and Cr-containing phases, structural homogenization of the layer, and the condition of the transition zone between the NiCrAl coating and the steel substrate.

### 2.7. Scratch Testing

The adhesion-related mechanical response of the coatings was evaluated by progressive-load scratch testing using a Revetest RST scratch tester (Anton Paar, Graz, Austria) equipped with a Rockwell diamond indenter (Anton Paar, Graz, Austria). During the test, the normal load was progressively increased while the friction force, acoustic emission, penetration depth, and residual depth were recorded. Critical loads corresponding to characteristic coating damage events were determined from the combined analysis of the recorded signals and optical inspection of the scratch track. In the present study, the third critical load, L_c3_, corresponding to severe coating damage and loss of interfacial integrity, was used as the principal comparative parameter. The L_c3_ values are reported in newtons and are considered scratch-adhesion indicators rather than direct tensile adhesion strength.

### 2.8. Surface Roughness Measurements

Surface roughness was characterized using a Profilm3D optical profilometer (Filmetrics, San Diego, CA, USA) based on non-contact optical profilometry. For each heat-treatment condition (800, 900, 1000, and 1100 °C), one representative coating specimen was selected for surface characterization. Five measurements were performed at different locations on the surface of each characterized specimen. The arithmetic mean roughness, Ra, was calculated from these five measurements, and the results are reported as mean ± standard deviation. Thus, the reported Ra values represent spatially repeated measurements on one characterized specimen for each heat-treatment condition rather than measurements from five independent specimens. The same measurement procedure was applied to all heat-treatment conditions to enable quantitative comparison of the temperature-dependent evolution of surface roughness.

### 2.9. Experimental Replication and Data Reporting

For each vacuum heat-treatment condition (800, 900, 1000, and 1100 °C), three coated specimens were prepared and treated under identical conditions. One specimen from each condition was selected for XRD, SEM, and EDS characterization, while the other two specimens were used for subsequent high-temperature oxidation and hot-corrosion experiments. Surface roughness was evaluated at five different locations on each characterized specimen, and the results are reported as the mean ± standard deviation. XRD, SEM, and EDS analyses were performed on one representative specimen for each temperature condition; accordingly, these results are discussed as representative structural and compositional observations.

## 3. Results and Discussion

### 3.1. Phase Evolution During Vacuum Heat Treatment

**Figure 3:**
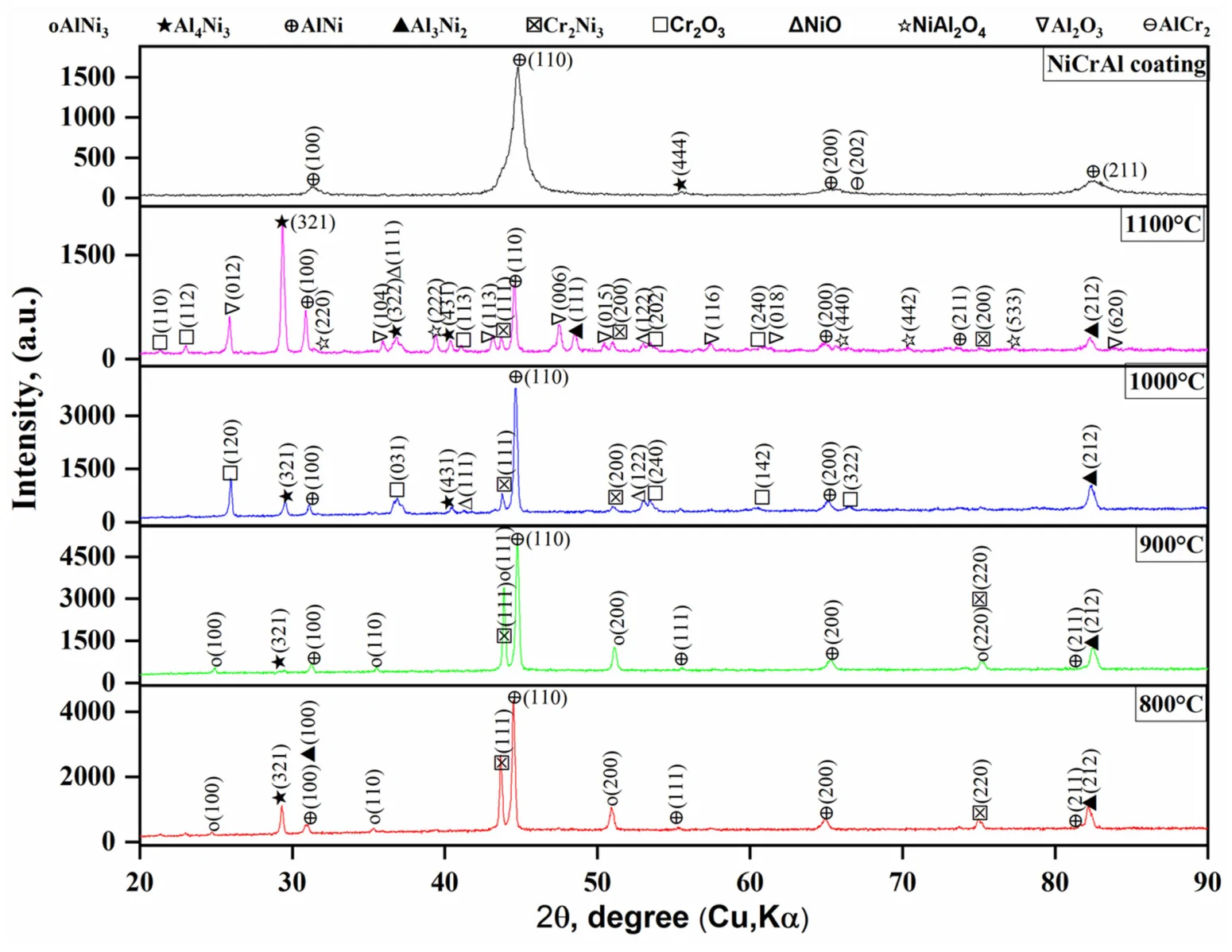
X-ray diffraction patterns of the detonation-sprayed NiCrAl coating in the as-sprayed state and after vacuum heat treatment at 800, 900, 1000, and 1100 °C for 4 h. The identified phases were assigned using the reference diffraction patterns listed in Table 4.

The X-ray diffraction patterns of the detonation-sprayed NiCrAl coating before and after vacuum heat treatment at 800, 900, 1000, and 1100 °C for 4 h indicate a complex and non-monotonic phase evolution of the material (Figure 3). The X-ray diffraction pattern of the as-sprayed NiCrAl coating is characterized by a relatively simple set of diffraction peaks. The most intense reflection at 2θ ≈ 44–45° was assigned to β-NiAl (110), in agreement with the corresponding reference pattern listed in Table 4. Additional reflections of this phase are observed at approximately 31°, 65°, and 82°, corresponding to the (100), (200), and (211) planes, respectively. The combination of these reflections allows us to conclude that one of the main crystalline constituents of the as-sprayed coating is the ordered β-NiAl phase with a B2 structure. β-NiAl is a characteristic phase of NiCrAlY and other MCrAlY coatings and plays a significant role in ensuring their high-temperature stability due to its ability to accommodate a substantial amount of Al [25,37]. The formation of β-NiAl in the as-sprayed detonation coating is in good agreement with the general understanding of the phase state of Ni–Cr–Al systems. In thermally sprayed NiCrAlY coatings, β-NiAl typically coexists with a Ni-rich γ-Ni(Cr,Al) constituent, and their ratio depends on the initial chemical composition and the thermal history of the particles during deposition [25,39]. For example, Zheng et al. showed that the as-sprayed HVOF-NiCrAlY coating is characterized by the presence of γ-NiCr and β-NiAl, whereas subsequent vacuum heat treatment changes the phase equilibrium and promotes the formation of γ′-Ni_3_Al [25].

A distinctive feature of the as-sprayed detonation coating is that it forms under highly non-equilibrium conditions. The particles undergo short-term intense heating, high-velocity impact with the substrate, and severe plastic deformation, followed by rapid cooling. Such a thermal history promotes the retention of a high dislocation density, microstrains, small coherent scattering domains, and local chemical heterogeneity. For detonation-sprayed NiCoCrAlY, Kala et al., using in situ high-temperature XRD, showed that subsequent heating is accompanied by a reduction in lattice strain and an increase in crystallite size, which the authors attributed to recovery and recrystallization processes in the initially highly defective structure [39]. Therefore, the intense and relatively broad AlNi(110) peak of the as-sprayed sample may reflect not only a significant fraction of β-NiAl but also a high degree of defectiveness of this phase. Therefore, subsequent changes in peak width and intensity after annealing should be associated simultaneously with changes in phase content, crystallite growth, reduction in microstrains, and possible changes in texture.

After vacuum heat treatment at 800 °C for 4 h, the phase composition becomes noticeably more complex. Along with the retained β-AlNi phase, reflections attributed to Al_4_Ni_3_, AlNi_3_, Al_3_Ni_2_, Cr_2_Ni_3_, and AlCr_2_ appear or become significantly more pronounced in the diffraction pattern. Thus, the relatively simple AlNi-dominated structure of the as-sprayed coating transforms into a pronounced multiphase state. This is one of the most significant results at this temperature, since it indicates that heat treatment already at 800 °C leads not only to the relaxation of crystal defects but also to an actual chemical redistribution of the components. The main reason for such restructuring is the activation of diffusion. A four-hour holding period at 800 °C creates conditions for the interdiffusion of Ni and Al and the formation of intermetallic compounds corresponding to different local compositions. The possibility of the simultaneous coexistence of several Ni–Al phases is in good agreement with experimental studies of the Al–Ni system. Urrutia et al., in their study of reactive diffusion in the Ni/Al system over the temperature range of 800–1170 °C, revealed the sequential formation of Al_3_Ni, Al_3_Ni_2_, Ni-poor AlNi, Ni-rich AlNi, and AlNi_3_ and showed that the growth of Al_3_Ni_2_ and AlNi_3_ is predominantly controlled by a diffusion mechanism [40]. Particularly pronounced after treatment at 800 °C is the reflection designated as Al_4_Ni_3_(321), located approximately in the 29–30° region. The increase in its intensity relative to the as-sprayed coating indicates intensive redistribution of Ni and Al. The presence of Cr_2_Ni_3_ and AlCr_2_ indicates that the phase transformation at 800 °C is not limited solely to the binary Ni–Al subsystem. Cr, which may partially exist in a supersaturated Ni-rich matrix after detonation spraying, can redistribute among different phases during annealing. In a more traditional description of NiCrAlY systems, the high-temperature structure is often considered a combination of γ-Ni, β-NiAl, γ′-Ni_3_Al, and α-Cr [34,41]. Overall, the state after treatment at 800 °C can be characterized as the initial stage of diffusion-induced redistribution and ordering of the initially non-equilibrium Ni–Cr–Al system.

As the temperature is increased to 900 °C, the next stage of phase evolution is observed. The peak at approximately 44–45°, attributed predominantly to AlNi(110) with a possible contribution from Cr_2_Ni_3_, remains dominant. At the same time, the relative prominence of some of the weaker intermetallic reflections changes. Unlike at 800 °C, where a large number of local Ni–Al states are formed, at 900 °C the increased diffusional mobility promotes further homogenization of the chemical composition and a transition from the formation of multiple intermediate phases toward more pronounced homogenization. Such behavior is in good agreement with the results of vacuum heat treatment of NiCrAlY coatings obtained using other deposition methods. In a study of a NiCrAlY coating on TC4, it was established that the as-sprayed layer contains γ-Ni, β-NiAl, and α-Cr; after vacuum heating, γ′-Ni_3_Al is formed, while at 900 °C the intensity of the γ′ reflections changes due to the rearrangement of the phase equilibrium [32]. Despite the differences in substrate and deposition technology, these results show that the temperature range around 900 °C is indeed sufficient for substantial redistribution of Ni–Al-containing phases. In addition, in situ high-temperature XRD studies of APS-NiCrAlY showed that heating causes gradual homogenization of the initially non-equilibrium structure and changes in the relative proportions of γ-Ni, γ′-Ni_3_Al, β-NiAl, and α-Cr. The authors emphasize that high-temperature exposure promotes chemical and structural homogenization of the thermally sprayed coating [41]. The state at 900 °C can most reasonably be characterized as a stage of intensive phase-diffusion homogenization, in which β-NiAl remains one of the main phases, while the system gradually approaches a more stable phase state.

To support the qualitative interpretation of the XRD data, a semi-quantitative comparison based on normalized integrated areas of selected characteristic reflections was performed (Table 5). Although these values do not represent absolute phase fractions, they provide useful diffraction-based indicators of the temperature-dependent phase evolution. The relative diffraction contribution assigned to β-NiAl remains dominant after heat treatment at 800–1000 °C, reaching approximately 81.7–88.1%, which confirms that this phase remains the principal crystalline constituent in this temperature range. At the same time, the contribution of Al_4_Ni_3_ is comparatively low at 900–1000 °C but increases markedly at 1100 °C to approximately 29.8%, indicating pronounced restructuring of the Ni–Al intermetallic subsystem at the highest treatment temperature. The relative contribution of AlNi_3_ also increases at 1100 °C, while Al_3_Ni_2_ remains comparatively minor under all conditions. Another important trend is the increase in the combined oxide/spinel contribution from 1.4–1.6% at 800–900 °C to 3.7% at 1000 °C and 8.5% at 1100 °C, which is consistent with the appearance and strengthening of oxide-related reflections in the diffraction patterns. Thus, the semi-quantitative analysis supports the conclusion that the 800–1000 °C range is characterized mainly by retention of β-NiAl with moderate redistribution among the intermetallic constituents, whereas treatment at 1100 °C produces the most pronounced multiphase restructuring accompanied by a clear increase in oxide/spinel-related diffraction features.

The values were obtained from normalized integrated areas of selected characteristic reflections and are presented only as semi-quantitative diffraction-based indicators. They do not represent absolute phase fractions because peak overlap, preferred orientation, and microstructural broadening were not accounted for by Rietveld refinement.

As the vacuum annealing temperature is increased to 1000 °C, the XRD pattern changes qualitatively. The intense AlNi(110) peak in the 44–45° region is still retained, confirming the stability of β-NiAl at this temperature. At 1000 °C, new reflections attributable to oxygen-containing phases, particularly NiO and Cr_2_O_3_, were observed; the phase assignments were made by comparison with the corresponding reference diffraction patterns listed in Table 4. Along with them, Ni–Al- and Ni–Cr-containing intermetallic constituents are retained. Therefore, the region around 1000 °C can be regarded as the onset of a transition from predominantly intermetallic restructuring to complex diffusion–phase–oxidation evolution. It should be noted that the absolute chamber pressure and residual gas composition were not measured during vacuum heat treatment. Consequently, the observed formation of oxide-containing phases cannot be quantitatively correlated with the oxygen partial pressure. Their appearance is therefore discussed in relation to the presence of residual oxidizing species in the furnace atmosphere and/or oxygen-containing constituents already present in the coating, rather than on the basis of a measured oxygen partial pressure. Under these conditions, residual oxygen- and water-containing species in the furnace atmosphere, together with oxygen already incorporated on powder-particle surfaces and within interlamellar oxide films formed during detonation spraying, may contribute to oxide formation. At 1000–1100 °C, increased diffusional mobility may promote the growth and crystallization of such oxygen-containing regions, making them detectable by XRD. Experimental evidence of related behavior in NiCrAlY systems under vacuum heat-treatment conditions was reported by Huang et al. Plasma-sprayed NiCrAlY samples were subjected to vacuum heat treatment in the temperature range of 1000–1100 °C; the authors observed the development of transient oxide products, including Al_2_O_3_, NiO, and Cr_2_O_3_, and demonstrated a significant effect of temperature and holding time on the formation of the surface oxide system [42]. Thus, the detection of Cr_2_O_3_ and NiO after treatment at 1000 °C in the investigated NiCrAl coating is consistent with the reported behavior of related MCrAlY systems.

With a further increase in temperature to 1100 °C, the most radical transformation of the diffraction pattern is observed. Unlike the states after treatment at 800–1000 °C, where the main AlNi(110) peak remained dominant, after treatment at 1100 °C, a large number of intense reflections appear over a wide 2θ range of approximately 20–70°. The reflection attributed to Al_4_Ni_3_(321) in the region around 29° becomes particularly pronounced, while Al_3_Ni_2_, AlNi, Cr_2_Ni_3_, Cr_2_O_3_, NiO, α-Al_2_O_3_, and the spinel NiAl_2_O_4_ phase are simultaneously detected, in accordance with the reference diffraction patterns listed in Table 4. This indicates that at 1100 °C the coating transitions from a stage of predominant homogenization to profound structural and phase reconstruction. This effect is primarily determined by the sharp increase in diffusion coefficients in accordance with the Arrhenius law. Increasing the temperature from 1000 to 1100 °C significantly increases the rates of bulk, grain-boundary, and interfacial diffusion of Ni, Al, and Cr. As a result, previously localized concentration inhomogeneities are actively redistributed, and the phase composition begins to be governed not so much by the inherited structure of individual detonation-sprayed particles as by the high-temperature thermodynamics of the system. At the same time, at 1100 °C, the role of the oxidation component increases significantly. For APS-NiCrAlY, during in situ heating to 1150 °C, Vijay et al. established that stable α-Al_2_O_3_ and spinel phases begin to be reliably detected above 1000 °C, simultaneously with the transformation of β-NiAl into γ′ and γ constituents [41]. This is almost identical to the trend observed in the present experiment: after treatment at 1100 °C, the intensity and number of oxide reflections increase sharply. The particularly significant feature is the formation of Al_2_O_3_. The strong affinity of Al for oxygen favors preferential formation of Al-containing oxides when oxygen is available. However, because the actual chamber pressure was not recorded, the oxygen activity during the present heat treatment cannot be quantified. Therefore, the formation of Al_2_O_3_ should be discussed in terms of the observed phase evolution rather than attributed to a specific vacuum level. Therefore, during prolonged heating, Al diffuses toward the regions most accessible to oxygen—the outer surface and interlamellar boundaries. As a result, aluminum is gradually depleted from β-NiAl and bound in Al_2_O_3_. For NiCrAlY coatings at 1100 °C, the transformation of metastable θ-Al_2_O_3_ into the more stable α-Al_2_O_3_ has previously been demonstrated, and this process is accompanied by intensive interdiffusion and changes in the phase composition of the metallic layer [43]. A related process is the formation of NiO. The appearance of NiO indicates that, locally, selective oxidation of Al alone is no longer sufficient to completely suppress the oxidation of Ni. This may occur in Ni-rich regions where the concentration of available Al is reduced due to its diffusional depletion. The formation of NiO, in turn, creates favorable conditions for the formation of NiAl_2_O_4_ spinel through its interaction with Al_2_O_3_. Thermodynamic analysis of the Ni–Al–O system performed by Kuo et al. showed that the stability of NiAl_2_O_4_ is determined by the activity of NiO and the partial pressure of oxygen; when the corresponding conditions are reached, the formation of nickel aluminate spinel is thermodynamically possible [44]. Therefore, the simultaneous detection of NiO, Al_2_O_3_, and NiAl_2_O_4_ after treatment at 1100 °C represents an internally consistent phase composition.

The origin of the oxide phases detected after vacuum heat treatment should be interpreted cautiously because the absolute chamber pressure and residual gas composition were not measured; therefore, the oxygen partial pressure cannot be reconstructed, and the individual oxidation pathways cannot be quantitatively separated. Nevertheless, several mechanisms are physically plausible. Residual oxygen- and water-containing species in the furnace atmosphere can provide sufficient oxygen activity for oxidation even under reduced-pressure conditions. Previous studies of NiCrAlY and CoNiCrAlY coatings have shown that NiO, Cr-rich oxides, alumina, and spinel-type oxides can form during heat treatment at 1000–1100 °C under vacuum or low-pressure conditions, while increasing temperature and holding time generally promote the development of a denser and more stable alumina-rich oxide layer [42,45]. In addition, oxygen already present on powder-particle surfaces or within interlamellar oxide films formed during thermal spraying may contribute to internal or interlamellar oxidation along splat boundaries, pores, and other fast-diffusion paths. Oxidation occurring exclusively during cooling is considered less likely because the specimens were furnace-cooled under vacuum; however, it cannot be completely excluded. The appearance of NiAl_2_O_4_ is also consistent with thermodynamic predictions for the Ni–Al–O system, where spinel formation depends on local NiO activity and oxygen partial pressure [44]. Accordingly, the detected Al_2_O_3_, Cr_2_O_3_, NiO, and NiAl_2_O_4_ phases are interpreted as the result of combined high-temperature oxidation processes involving residual oxidizing species in the furnace atmosphere and pre-existing oxygen-containing constituents in the coating, rather than as evidence of a single uniquely identified oxidation mechanism.

X-ray photoelectron spectroscopy (XPS) was not performed in the present study; therefore, the oxidation states of Al, Cr, and Ni were not independently verified by surface-sensitive chemical-state analysis. The identification of Al_2_O_3_, Cr_2_O_3_, NiO, and NiAl_2_O_4_ was based primarily on XRD peak matching using the reference diffraction cards listed in Table 4 and was supported by the corresponding EDS elemental distributions. Published XPS/XRD studies of Ni–Cr–Al alloys have demonstrated the formation of α-Al_2_O_3_, Cr_2_O_3_, and smaller amounts of NiO during high-temperature oxidation, with the resulting oxide sequence being strongly dependent on oxygen partial pressure [46]. In addition, high-resolution XPS investigations of oxidized NiAl have independently demonstrated the formation of NiAl_2_O_4_ spinel [47]. Accordingly, the oxide assignments in the present study should be regarded as XRD-based phase identifications supported by EDS observations and literature evidence rather than as direct XPS confirmation of the oxidation states.

### 3.2. Surface Morphology and Roughness Evolution

**Figure 4:**
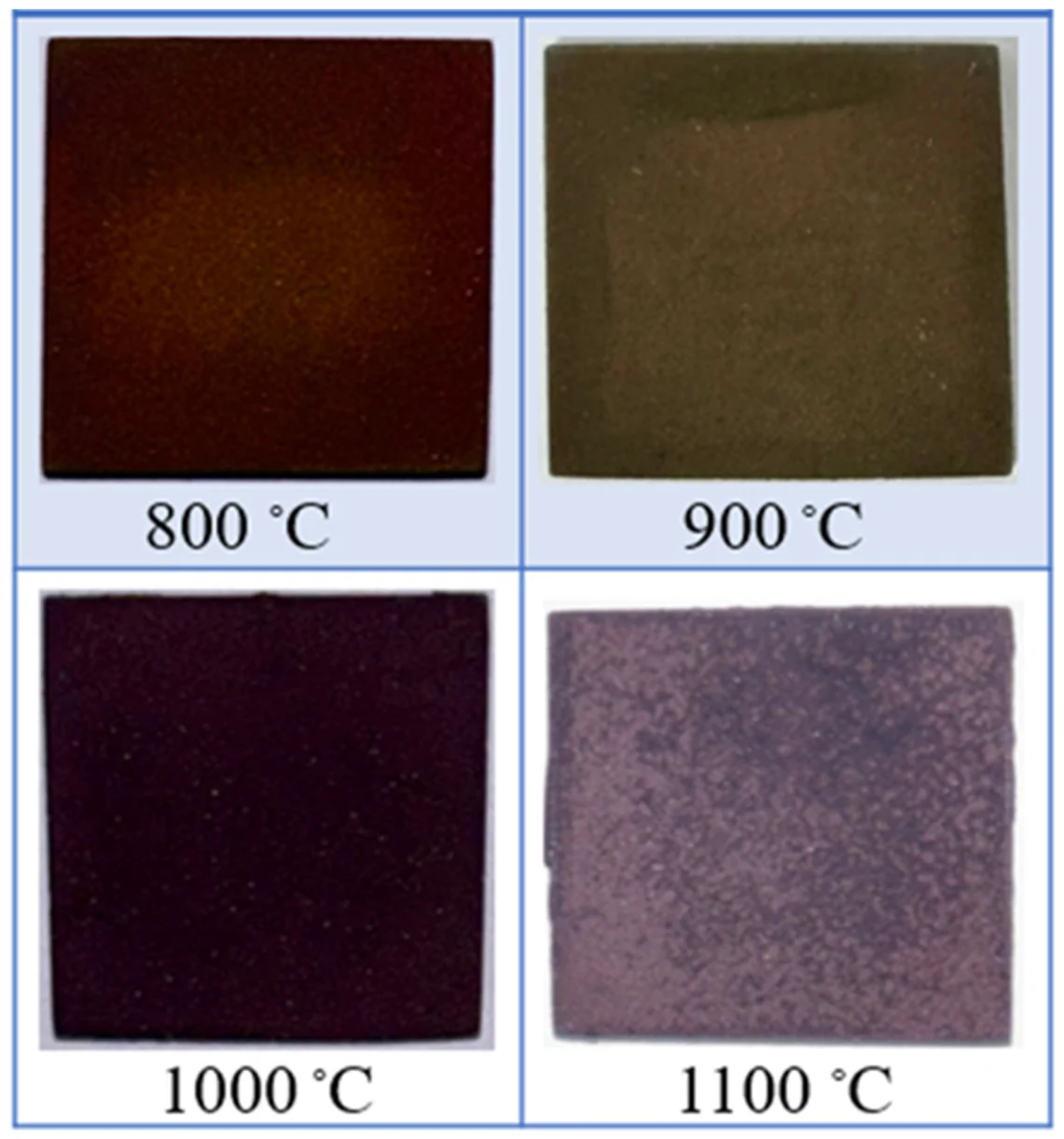
Surface appearance and roughness evolution of detonation-sprayed NiCrAl coatings after vacuum heat treatment at 800, 900, 1000, and 1100 °C for 4 h.

Vacuum heat treatment leads to a noticeable change in the appearance of detonation-sprayed NiCrAl coatings (Figure 4). Even a visual comparison of the samples shows that with increasing temperature, not only the surface color but also its optical uniformity changes. These changes reflect a combination of processes occurring in the surface layer: diffusional redistribution of Ni, Al, and Cr, restructuring of Ni–Al intermetallic phases, relaxation of the initial structure of the sprayed coating, and formation of thin oxygen-containing surface layers. For MCrAlY coatings, it is known that the surface condition and roughness significantly influence the morphology and kinetics of surface oxide formation at 800–1100 °C [48]. The quantitative surface roughness results obtained after vacuum heat treatment are summarized in Table 6.

The surface roughness exhibited a non-monotonic dependence on heat-treatment temperature. The Ra value decreased from 6 ± 0.20 μm at 800 °C to 5.3 ± 0.18 μm at 900 °C and remained at a comparable level of 5.3 ± 0.19 μm at 1000 °C. At 1100 °C, Ra increased to 5.9 ± 0.21 μm. Thus, the lowest surface roughness was observed after heat treatment at 900 °C. The subsequent increase at 1100 °C is consistent with the visually more heterogeneous and locally mottled surface morphology observed at the highest treatment temperature.

After treatment at 800 °C, the surface has a dark brown, locally reddish-brown hue and appears relatively uniform. Such a color already differs noticeably from the typical metallic appearance of the as-sprayed NiCrAl coating and indicates transformation of the uppermost surface layer. For the sample treated at 800 °C, a surface roughness value of Ra = 6 ± 0.20 μm was obtained. This relatively high roughness indicates that the pronounced surface topography formed during detonation spraying is retained. Such a surface is formed as a result of the successive deposition of molten and partially molten particles, their flattening, and overlapping. Therefore, a four-hour holding period at 800 °C already causes noticeable phase changes. This is in good agreement with the concepts of solid-state heat treatment of thermal-sprayed coatings. Since 800 °C is significantly below the melting temperature of the main Ni–Al phases, smoothing occurs not due to surface melting but exclusively through surface and grain-boundary diffusion. First, the finest irregularities and contact zones between adjacent particles are modified, while larger protrusions formed by individual splats are retained.

At 900 °C, the surface acquires a gray-brown, locally olive hue and visually appears somewhat more uniform. Compared with the sample treated at 800 °C, the intensity of the reddish-brown coloration decreases. The sample treated at 1000 °C visually differs significantly from those treated under the first two conditions. Its surface becomes the darkest—burgundy-brown or dark purple. This change is particularly interesting in combination with the XRD results, since it is at 1000 °C that oxygen-containing phases, including NiO and Cr_2_O_3_, begin to appear clearly in the diffraction pattern. For NiCrAlY, vacuum treatment in the temperature range of 1000–1100 °C indeed leads to the formation and transformation of surface oxides. Zheng et al. showed that after VHT at 1000 and 1100 °C, the NiCrAlY surface becomes covered with granular oxide products, while XRD confirms the presence of Cr_2_O_3_ and α-Al_2_O_3_ [25]. Keller et al. also established that the surface composition of MCrAlY after vacuum treatment depends on the temperature, pressure in the vacuum system, and oxygen content in the initial coating [26]. Therefore, the pronounced color change at 1000 °C is in good agreement with the onset of more pronounced oxide transformation. The mean roughness decreased from 5.95 ± 0.20 μm at 800 °C to 5.3 ± 0.19 μm at 1000 °C, corresponding to a reduction of approximately 10.2%.

From an engineering perspective, changes in surface roughness and morphology may be important beyond their purely geometrical characterization, because surface topography can influence real contact conditions, lubricant retention, local heat transfer, friction, and wear. Recent studies on deliberately engineered surface textures have demonstrated that controlled modification of surface morphology can significantly affect thermal and tribological performance. For example, Dai et al. showed that tailored micro-texturing of aviation gear surfaces improved heat dissipation and reduced wear under selected lubrication conditions, demonstrating that surface morphology can act as an important functional design parameter rather than merely a geometrical surface characteristic [49]. In the present NiCrAl coatings, the observed decrease in Ra and the temperature-induced modification of surface morphology therefore indicate that vacuum heat treatment may also modify the functional response of the surface. However, because tribological and direct thermal-performance tests were not performed in the present study, such implications should be regarded as prospective rather than experimentally demonstrated.

After treatment at 1100 °C, the coating color becomes significantly lighter—gray-pink or gray-violet—and the surface simultaneously appears more mottled. This state differs fundamentally from the dark coating after treatment at 1000 °C. It is at 1100 °C that the most complex set of oxygen-containing phases, including Al_2_O_3_, Cr_2_O_3_, NiO, and NiAl_2_O_4_, is observed, simultaneously with substantial redistribution of the Ni–Al intermetallic phases. Therefore, the surface becomes multiphase and chemically heterogeneous. For HVOF-NiCrAlY after vacuum treatment at 1100 °C, Zheng et al. observed a pronounced surface restructuring: γ′-Ni_3_Al transformed from a granular to a lamellar morphology, porosity decreased, and after subsequent high-temperature exposure, a predominantly α-Al_2_O_3_-containing surface layer was formed [25]. At the same time, studies of MCrAlY coatings show that even small differences in roughness significantly affect the morphology of the oxide film: its growth conditions differ in valleys and on asperities, causing a rough surface to form a more heterogeneous oxide layer than a mechanically smoothed surface [48]. The observed mottling does not necessarily indicate coating failure or delamination. It may be associated with differences in the thickness of oxide products on the peaks and in the valleys of the initial detonation-sprayed surface relief, as well as with local variations in Al and Cr contents. Thus, the temperature-induced evolution of surface morphology may have practical implications for subsequent contact and heat-transfer behavior, although these effects require direct tribological and thermal verification.

### 3.3. Microstructural and Elemental Evolution

**Figure 5:**
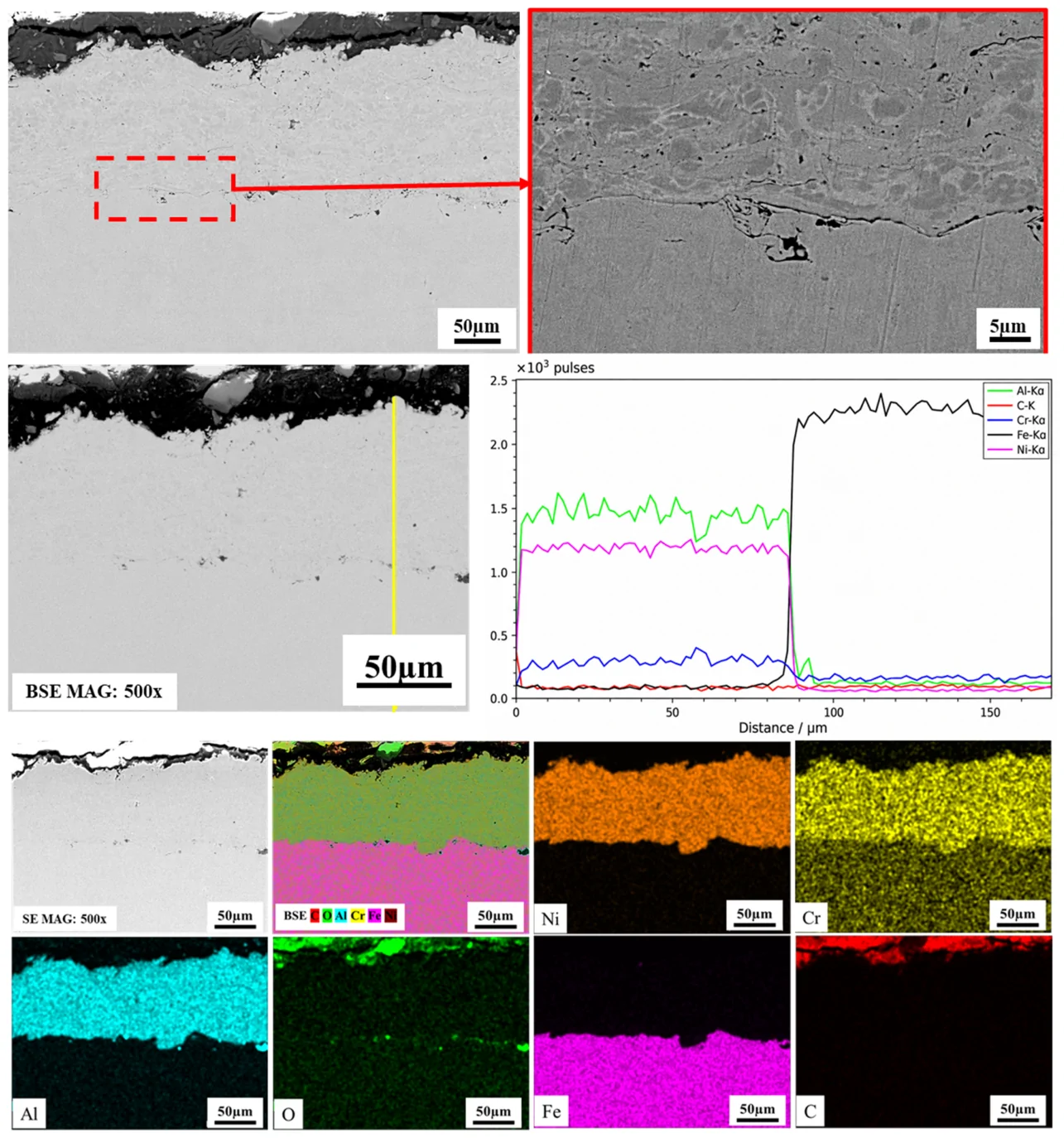
Cross-sectional microstructure and elemental distribution of the as-sprayed detonation-sprayed NiCrAl coating.

Figure 5 shows the cross-sectional microstructure of the NiCrAl coating immediately after detonation spraying, i.e., before vacuum heat treatment. Combined analysis of the BSE images, linear EDS profile, and elemental distribution maps shows the formation of a relatively dense and continuous coating layer with a thickness of approximately 85–90 μm. The interface between the coating and the steel substrate is clearly distinguishable both by BSE contrast and by the sharp change in elemental composition. Ni, Cr, and Al predominate in the coating region, whereas after crossing the interface, the intensities of these elements decrease sharply and Fe begins to dominate. This pattern indicates the preservation of a relatively distinct coating–substrate interface immediately after spraying and the absence of a developed diffusion transition zone. During detonation spraying, the particles are accelerated to high velocities, heated, and undergo intense deformation upon impact with the substrate; however, the duration of thermal exposure is extremely short. Therefore, coating formation is governed primarily by mechanical interlocking and local interaction of highly deformed particles, while the time available for significant bulk diffusion of Ni, Cr, Al, and Fe is insufficient. A similar dense splat-like structure was described for detonation-sprayed NiCrAlY coatings by Kamal et al., who reported a dense, continuous structure with a small amount of residual porosity localized predominantly along splat boundaries [10].

At ×2000 magnification, individual elongated dark discontinuities and boundaries between deformed particles become visible within the coating. They do not form a developed network of through-thickness cracks; however, they indicate the retention of the lamellar nature of the initial detonation-sprayed layer. Such regions may correspond to inter-splat pores, incomplete metallurgical contact between adjacent particles, or local oxide interlayers. For NiCrAlY coatings produced by detonation spraying, similar oxide regions were indeed observed along splat boundaries; their formation was attributed to the oxidation of Al during particle flight and deposition. Therefore, the presence of a small amount of dark interlamellar regions in this coating should be considered a natural consequence of its formation mechanism rather than an indication of coating failure [50]. The linear EDS profile is particularly informative. Within the first approximately 85–90 μm, the concentration profiles of Ni, Al, and Cr vary relatively little, indicating a fairly uniform distribution of the main components throughout the coating thickness at the scale of this measurement. At the interface with the substrate, the contents of Ni, Al, and Cr decrease sharply, while the Fe signal simultaneously increases abruptly. This allows us to conclude that, after detonation spraying, interdiffusion between the coating and the substrate is still limited. That is, the initial interface is predominantly mechanical–metallurgical in nature and has not yet transformed into a broad diffusion zone. This feature is expected to change during subsequent vacuum heat treatment, as increasing the temperature to 800–1100 °C sharply enhances the diffusional mobility of the elements.

The elemental distribution maps confirm the results of the line analysis. Ni and Al are predominantly localized in the coating, whereas Fe is almost entirely concentrated in the substrate. Cr is also predominantly associated with the coating layer. At the same time, no large regions of complete chemical segregation are observed within the coating, indicating sufficiently effective mixing of the initial components during the formation of the detonation-sprayed layer. However, the absence of large-scale segregation does not necessarily indicate that phase equilibrium has been achieved. On the contrary, the high-velocity detonation process is characterized by the formation of a chemically relatively homogeneous but structurally and phase-wise non-equilibrium state. This is in good agreement with the X-ray phase analysis results of the as-sprayed coating. For detonation-sprayed Ni–Cr–Al systems, it has previously been established that, depending on the energy parameters of the spraying process, the phase composition can differ significantly from that of the initial powder material. Rakhadilov et al. showed that at a relatively high degree of barrel filling, predominantly Ni–Cr-containing phases were formed in the coatings, whereas Ni–Al phases became pronounced only when the thermal load was reduced and Al retention increased [9]. This is particularly important for interpreting the present coating: even despite the presence of a noticeable amount of Al according to EDS, not all aluminum necessarily exists as a separate crystalline β-NiAl phase detectable by XRD. The presented EDS maps directly confirm the presence of Al throughout almost the entire thickness of the coating. Therefore, some of the Al may be present in solid solution, in very finely dispersed Ni–Al regions, in interlamellar oxide layers, or in a structurally disordered state. Such a situation is typical of rapidly solidified thermal-sprayed coatings, since XRD primarily detects sufficiently large and well-crystallized phases. In the study by Fritscher and Lee on a NiCoCrAlY coating, it was also shown that the phase composition immediately after spraying differs noticeably from the fully thermally stabilized state: in the as-sprayed condition, γ-Ni, β-NiAl, and γ′-Ni_3_Al were detected by XRD, but the relative content of the γ phase was significantly higher than after subsequent structural stabilization [51]. Although the LPPS technology used by the authors differs from detonation spraying, this result demonstrates a fundamentally important principle: a rapidly formed coating does not necessarily exhibit a phase ratio corresponding to the equilibrium state of a given chemical composition. In addition, the structure of the as-sprayed coating is in a highly deformed state. During detonation spraying, the particles impact the surface at high velocity and undergo intense plastic deformation. This leads to an increased dislocation density, local microstrains, and crystallite refinement. Therefore, the diffraction peaks of the as-sprayed coating may be relatively broad and exhibit lower intensity than after high-temperature annealing.

**Figure 6:**
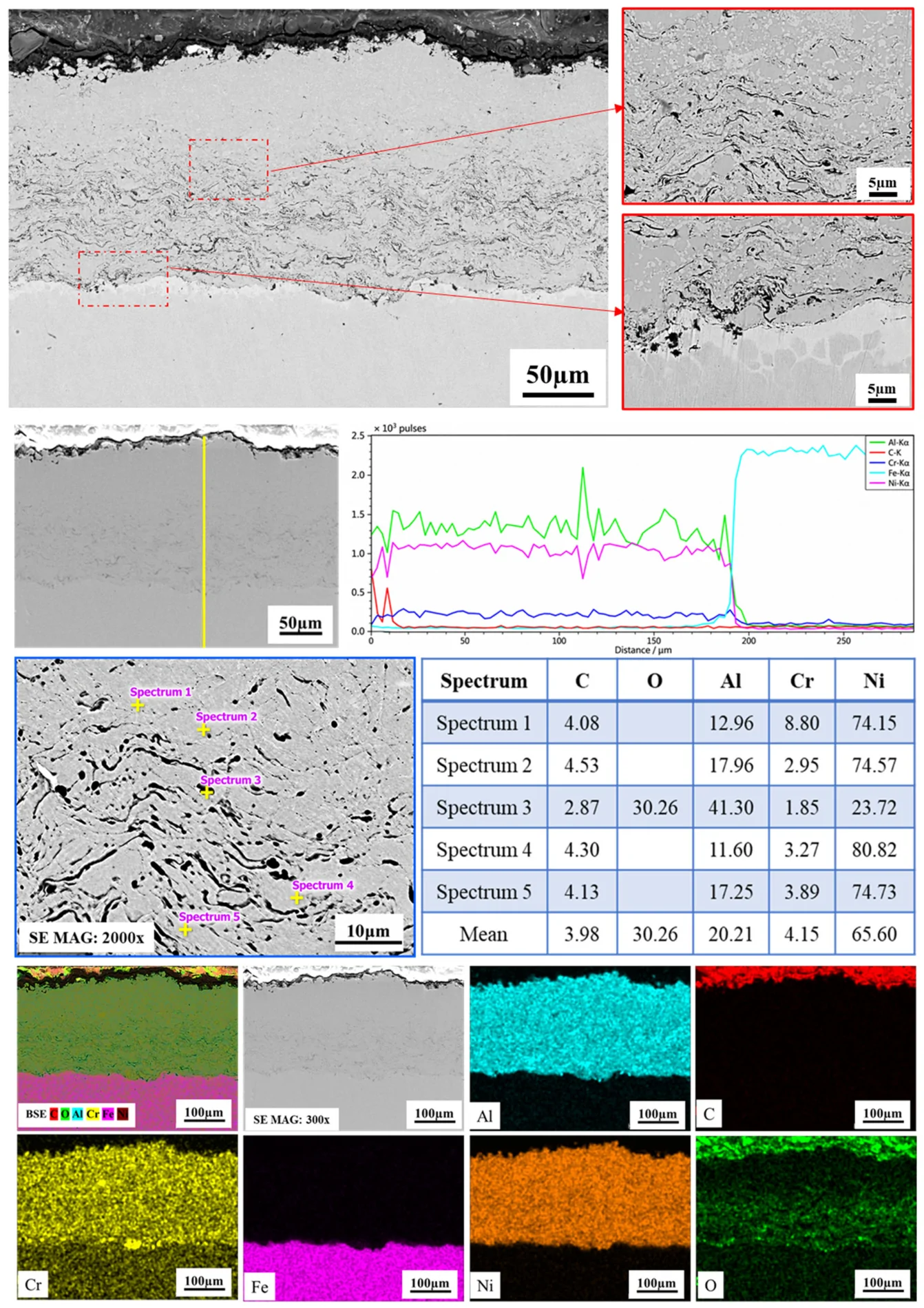
Microstructure and elemental composition of the detonation-sprayed NiCrAl coating after vacuum heat treatment at 800 °C for 4 h.

After vacuum heat treatment at 800 °C for 4 h, the microstructure of the detonation-sprayed NiCrAl coating undergoes noticeable changes compared with the as-sprayed state (Figure 6). At the same time, the coating generally retains its integrity and continuity through the thickness; however, structural heterogeneity within the layer becomes significantly more pronounced. In the cross-sectional BSE image, elongated dark regions oriented predominantly parallel to the surface and the substrate interface are clearly distinguishable. Such morphology corresponds to the inherited lamellar structure of the detonation-sprayed coating and indicates that the inter-splat boundaries do not completely disappear at 800 °C. In the as-sprayed detonation state, the coating exhibited relatively uniform BSE contrast, while individual interlamellar discontinuities were localized predominantly at the boundaries of the deformed particles. After holding at 800 °C, these boundaries become more distinct and, in some regions, form extended tortuous features. Therefore, the structural changes at this temperature cannot be interpreted solely as coating densification.

At the early stage of heat treatment, both diffusion-induced improvement of contact between individual splats and the manifestation of pre-existing interparticle heterogeneities may occur simultaneously due to phase redistribution and differences in the BSE contrast of the forming phases. The enlarged region highlighted by the red frame is particularly informative. A pronounced fibrous-lamellar structure consisting of light and darker elongated regions is observed in this area. Such contrast should not automatically be interpreted as porosity. In BSE mode, brightness is determined mainly by the average atomic number of the local region: Ni-rich areas appear brighter, whereas Al-rich and especially Al–O-containing regions may exhibit darker contrast. Therefore, some of the observed dark “interlayers” represent not voids, but chemically distinct products resulting from elemental redistribution. This is directly confirmed by local EDS analysis. In spectra 1, 2, 4, and 5, Ni remains predominant: its content is approximately 74–81%, with Al contents of about 11.6–18.0% and Cr contents of about 3–9%. Such a composition corresponds to the Ni-rich metallic matrix of the coating. At the same time, spectrum 3 differs sharply from the others: it contains approximately 30.26% O, 41.30% Al, 23.72% Ni, and only a small amount of Cr. This is an important result, as it indicates the presence of local Al–O-rich regions after heat treatment at 800 °C. According to the elemental maps, Ni occupies nearly the entire volume of the coating and forms a continuous metallic matrix. Al is also distributed throughout the entire thickness of the layer; however, regions with locally increased Al concentration are observed. Cr is present predominantly within the coating and exhibits a less uniform distribution than Ni. Fe, in contrast, is almost entirely concentrated in the substrate. Thus, even after 4 h at 800 °C, the NiCrAl coating–steel substrate interface remains relatively sharp. Thus, 800 °C represents an early stage of heat-induced redistribution in the present coating, at which the inherited detonation-sprayed architecture is still retained, and local chemical heterogeneity develops before pronounced coating–substrate interdiffusion occurs. This behavior is qualitatively consistent with the early-stage restructuring reported for thermally treated NiCrAlY/MCrAlY coatings, although direct equivalence is not expected because of differences in composition and deposition technology.

**Figure 7:**
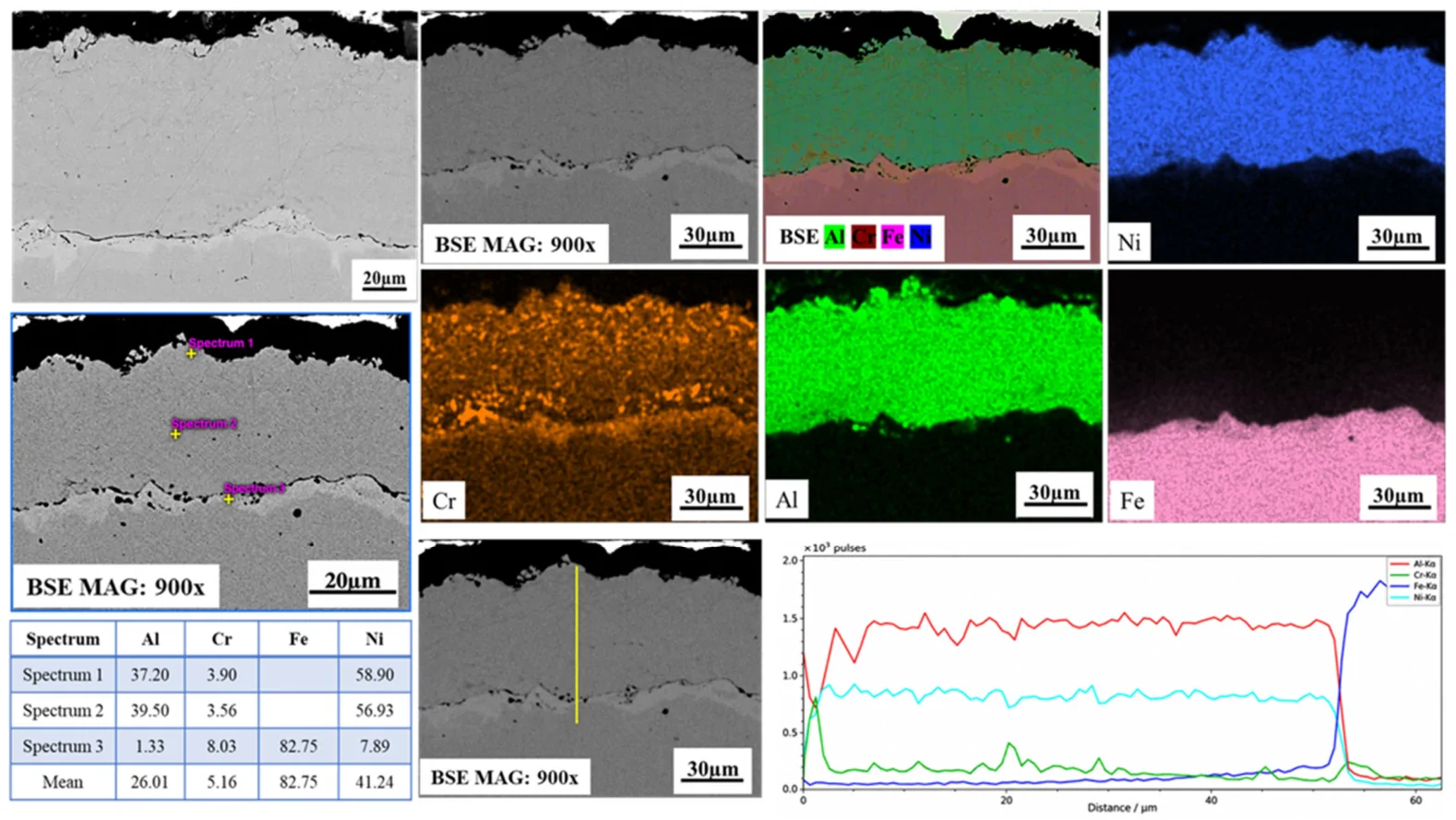
Microstructure and elemental distribution in the detonation-sprayed NiCrAl coating after vacuum heat treatment at 900 °C for 4 h.

After vacuum heat treatment at 900 °C for 4 h, the detonation-sprayed NiCrAl coating exhibits more pronounced structural restructuring compared with the state after treatment at 800 °C (Figure 7). In the presented BSE image, the coating retains continuity throughout the investigated region, while the interface with the substrate remains clearly distinguishable. At the same time, the microstructure of the layer becomes visually more homogeneous: the well-developed system of elongated dark interlamellar regions characteristic of the 800 °C condition is noticeably less pronounced. This suggests that increasing the temperature from 800 to 900 °C activates diffusion-driven compositional homogenization and solid-state sintering of adjacent splats. In the cross-section, the main part of the coating is characterized by a relatively uniform gray BSE contrast. Individual dark pores and elongated defects are present predominantly near the coating–substrate interface, whereas they are significantly less pronounced in the central and near-surface regions. Importantly, most of these discontinuities do not form a continuous network through the coating thickness. Therefore, vacuum heating at 900 °C does not cause macroscopic failure or delamination of the coating but, on the contrary, contributes to maintaining its structural integrity.

The state of the region near the interface is particularly interesting. In the SEM image, a chain of dark elongated regions and individual nearly spherical pores is observed along the coating–substrate interface. They are localized predominantly directly above or near the interface. Such localization may indicate that the coating–substrate interface remains the most structurally heterogeneous region after treatment at 900 °C. However, these dark regions cannot be unequivocally interpreted as pores. BSE contrast is determined by the average atomic number; therefore, Al-rich regions may also appear darker than the Ni-rich matrix. Nevertheless, the nearly black rounded regions with sharp boundaries most likely correspond to actual micropores or cavities. Elongated, less dark regions may additionally include areas with different phase compositions. Local EDS analysis makes it possible to substantially refine this interpretation. In spectrum 1, approximately 37.20% Al, 3.90% Cr, and 58.90% Ni were determined, whereas in spectrum 2, the corresponding values were 39.50% Al, 3.56% Cr, and 56.93% Ni. Thus, both points within the coating correspond to pronounced Ni–Al-rich regions with a relatively low Cr content. This fundamentally differs from the pattern observed after treatment at 800 °C, where a high oxygen content and pronounced chemical heterogeneity were detected in certain regions. In the presented EDS analysis of the sample after treatment at 900 °C, oxygen is absent among the quantitatively determined components. Therefore, at least at the investigated points, the structure is determined predominantly by the redistribution of Ni–Al–Cr rather than by the formation of large oxygen-containing regions. More convincing data are provided by the linear EDS profile. Throughout most of the coating, the Al and Ni contents remain relatively stable, after which their intensities decrease sharply near the interface, simultaneously with a rapid increase in the Fe signal. This shows that after treatment at 900 °C for 4 h, a developed broad interdiffusion zone has not yet formed. At the same time, the transition becomes somewhat more structurally complex than in the as-sprayed state, corresponding to the initial stage of chemical interaction with the substrate.

The elemental maps show that Ni forms a continuous coating layer with a relatively uniform distribution throughout the thickness. Al is also distributed throughout almost the entire coating but exhibits more pronounced local heterogeneity. Cr is distributed significantly less uniformly and exhibits individual Cr-enriched regions. Fe is almost entirely localized in the substrate. The Cr map is of particular interest. In the lower part of the coating, directly near the interface, regions with an increased Cr signal are noticeable. This may indicate thermally induced redistribution of Cr from the Ni-rich matrix. With increasing temperature, the solubility and chemical potential of Cr in different Ni–Al phases change, making the local formation of Cr-rich regions or secondary Cr-containing phases possible. The structural evolution observed at 900 °C is broadly consistent with published observations for thermally treated NiCrAlY/MCrAlY coatings, where heating promotes redistribution of Ni–Al-containing phases and progressive chemical homogenization. However, the comparison is qualitative because the present coating has a higher Al content, a lower Cr content, contains no Y, and was produced by detonation spraying rather than by conventional APS or HVOF routes.

**Figure 8:**
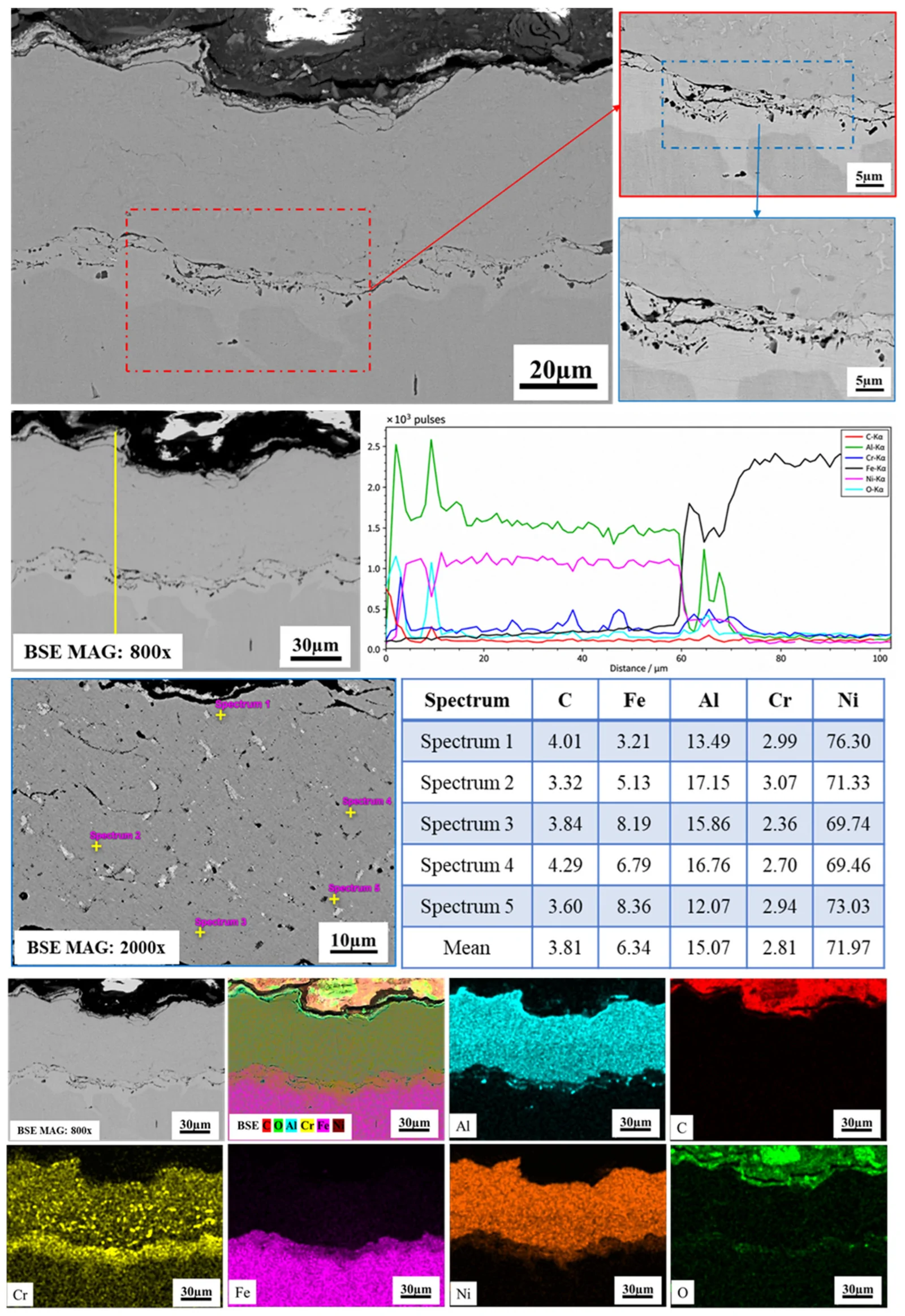
Microstructure and elemental distribution of the detonation-sprayed NiCrAl coating after vacuum heat treatment at 1000 °C for 4 h.

After vacuum heat treatment at 1000 °C for 4 h, the structure of the detonation-sprayed NiCrAl coating changes much more profoundly than after treatment at 800 and 900 °C (Figure 8). According to the presented BSE images, the coating retains its continuity and overall integrity; however, the pronounced lamellar heterogeneity characteristic of detonation spraying is almost no longer observed within the layer. The main part of the coating exhibits a relatively uniform gray contrast, whereas the most noticeable defects and chemically contrasting regions are concentrated mainly near the interface with the substrate.

In the enlarged SEM images, only isolated small dark inclusions and pores are visible within the main part of the coating. This is an important indication of the transition from simple thermal homogenization of the coating to pronounced interdiffusional interaction between the coating and the substrate. Local EDS analysis of the investigated sample shows that the main part of the coating remains Ni-rich after treatment at 1000 °C. For spectra 1–5, the Ni content is approximately in the range of 69.46–76.30%, Al 12.07–17.15%, Cr 2.36–3.07%, whereas Fe is approximately 3.21–8.36%. The average values for the selected points are approximately 71.97% Ni, 15.07% Al, 2.81% Cr, and 6.34% Fe. The linear EDS profile shows that the Ni and Al signals remain relatively stable throughout most of the coating; however, in the region of approximately 60–70 μm, the transition is not abrupt but more complex: the Fe content begins to increase before the signals of the coating components completely decrease. This type of concentration profile is characteristic of a developing interdiffusion zone. According to the elemental maps, Ni and Al remain concentrated predominantly in the coating, whereas Fe is concentrated in the substrate. However, near the interface, the distribution contours of these elements already partially overlap. The chemical heterogeneity of Cr is particularly noticeable: its content in the bulk of the coating is low, but the signal increases in certain interfacial and near-surface regions. Such localization of Cr may be associated with a decrease in its solubility in certain Ni–Al phases as the system approaches an equilibrium state.

The pronounced structural homogenization observed at 1000 °C is consistent with the general trend reported for vacuum-treated NiCrAlY/MCrAlY coatings, in which elevated-temperature exposure promotes redistribution of Ni–Al phases and reduction in the inherited heterogeneity of the sprayed structure [25]. Nevertheless, because the present NiCrAl coating differs in both composition and deposition route, this comparison is used only to provide qualitative context rather than to imply direct microstructural equivalence.

**Figure 9:**
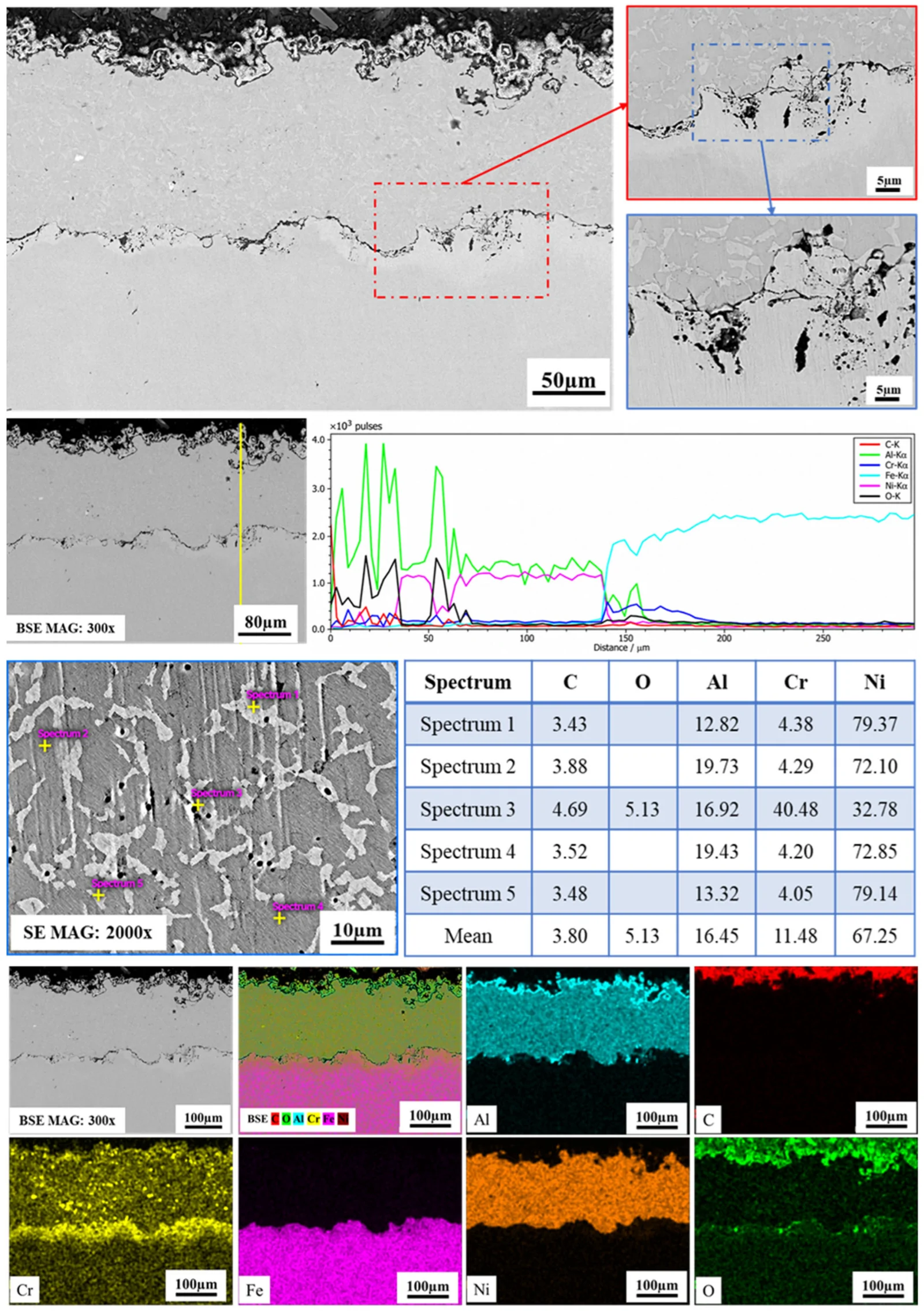
Microstructure and elemental distribution in the detonation-sprayed NiCrAl coating after vacuum heat treatment at 1100 °C for 4 h.

After vacuum heat treatment at 1100 °C for 4 h, the detonation-sprayed NiCrAl coating reaches the most profound degree of structural transformation among the investigated conditions of 800–1100 °C (Figure 9). The presented SEM/BSE images show that the bulk of the coating remains continuous; however, the nature of the coating–substrate interface and the near-surface region becomes significantly more complex. If at 800–900 °C the structural changes were mainly associated with internal homogenization of the lamellar layer, and at 1000 °C interdiffusion was already clearly manifested, then at 1100 °C diffusion interaction becomes one of the dominant mechanisms governing the evolution of the entire system. The main part of the coating appears relatively homogeneous in the BSE image and significantly less lamellar than in the as-sprayed detonation state. The boundaries of individual splats are barely distinguishable in the central part of the layer. This indicates the intensive development of recovery, recrystallization, crystallite growth, and interlamellar mass transfer processes. Such a pattern is expected, since increasing the temperature to 1100 °C sharply increases the diffusion coefficients of the components of the Ni–Cr–Al system. With a four-hour holding period, atomic mass transfer is no longer limited to short diffusion paths along defects and splat boundaries but also occurs through bulk diffusion. At 1100 °C, a nearly continuous metallic matrix is observed in the central part of the coating without a pronounced system of inter-splat gaps. This can be interpreted as a result of solid-state sintering and diffusion-induced “healing” of a significant portion of the initial interlamellar boundaries.

The most significant changes after treatment at 1100 °C are observed not in the central part of the coating but in the coating–substrate interfacial region. The interface becomes substantially more irregular and chemically heterogeneous. The enlarged SEM images reveal regions of complex contrast, individual microvoids, and local distortion of the initially relatively distinct coating–steel boundary. This morphology differs from that observed at 800 °C, where the interface remained predominantly sharp, and at 900 °C, where only local modification of the interfacial region was evident. At 1000 °C, Fe penetration into the coating becomes detectable, indicating the development of coating–substrate interdiffusion. After heat treatment at 1100 °C, this process becomes more pronounced, as evidenced by the broadened transition region and redistribution of Fe, Ni, Al, and Cr across the coating–substrate interface.

The EDS line scan across the coating–substrate interface provides additional evidence of elemental redistribution (Figure 9). Along the analyzed line, the coating region is characterized by dominant Ni and Al signals and a comparatively low Fe signal. On approaching the coating–substrate interface, located at approximately 140 μm along the line-scan coordinate, the Fe signal increases sharply, while the Ni and Al signals decrease substantially. Importantly, the value of approximately 140 μm represents the position of the interface along the selected EDS scan line and should not be interpreted as the coating thickness or as the Fe penetration depth. The elemental signals change over a finite transition region rather than through an ideally abrupt step, indicating a chemically broadened coating–substrate interface. Cr also exhibits local variations in this region, consistent with the chemical heterogeneity observed in the corresponding EDS maps. Because the line-scan data are expressed as X-ray pulse intensities rather than calibrated concentrations in wt.% or at.%, the profiles are interpreted semi-quantitatively and are used to demonstrate the spatial direction and relative extent of elemental redistribution rather than absolute concentration gradients or diffusion depths. Point EDS analysis further confirms the pronounced local compositional heterogeneity after treatment at 1100 °C. Spectrum 3 contained approximately 5.13 wt.% Fe, 16.92 wt.% Al, 40.48 wt.% Cr, and 32.78 wt.% Ni, whereas the Ni-rich regions represented by Spectra 1 and 5 contained approximately 79 wt.% Ni, 12.82–13.32 wt.% Al, and 4.05–4.38 wt.% Cr, with no Fe reported in these spectra. These results are consistent with substantial local redistribution of the constituent elements at 1100 °C. The relationship between the 4 h holding time and the coating thickness should also be considered when interpreting the observed interdiffusion. For diffusion-controlled redistribution, the characteristic diffusion distance can be approximated as L∼$\sqrt{Dt}$, where *D* is the temperature- and composition-dependent diffusion coefficient and *t* is the holding time. Studies of β-NiAl and multicomponent β-NiAl systems have demonstrated a strong increase in interdiffusion with temperature and a pronounced dependence on alloy composition [52,53]. In the present study, the coating thickness was approximately 85–90 μm, while the SEM/EDS observations show that the most pronounced compositional changes after 4 h are concentrated in the coating–substrate interfacial region rather than uniformly distributed throughout the entire coating. The comparatively distinct interfaces observed at 800–900 °C and the progressively broader interdiffusion features at 1000–1100 °C are consistent with this temperature-dependent diffusion behavior. Similar development of an interdiffusion zone after vacuum heat treatment at 1000 °C for 4 h has been reported for NiCrAlY-based coating systems [33]. Thus, the selected 4 h holding time was sufficient to reveal temperature-dependent interfacial redistribution while preserving a substantial portion of the original coating structure.

The EDS results further demonstrate the chemically heterogeneous character of the coating after vacuum heat treatment at 1100 °C. Most analyzed regions within the coating remain Ni-rich. For example, Spectrum 1 contains approximately 79.37 wt.% Ni, 12.82 wt.% Al, and 4.38 wt.% Cr, while Spectrum 5 shows a similar composition with approximately 79.14 wt.% Ni, 13.32 wt.% Al, and 4.05 wt.% Cr. In contrast, Spectrum 3 exhibits a markedly different local composition, containing approximately 32.78 wt.% Ni, 16.92 wt.% Al, 40.48 wt.% Cr, and 5.13 wt.% Fe. This pronounced enrichment in Cr indicates substantial local elemental redistribution after heat treatment at 1100 °C rather than a chemically uniform transformation of the entire coating. The line-scan profile across the coating–substrate interface also shows a clear redistribution of the principal elements. Ni and Al dominate within the coating, whereas the Fe signal increases strongly upon entering the substrate. In the interfacial region, simultaneous changes in the Ni, Al, Cr, and Fe signals indicate the development of a chemically broadened transition zone. The elemental maps support this interpretation: Ni and Al remain predominantly localized within the coating, Fe is concentrated in the steel substrate, while Cr exhibits a more heterogeneous distribution with locally enriched regions. Thus, the microstructural evolution at 1100 °C is characterized by both extensive homogenization of the bulk coating and localized chemical redistribution near the coating–substrate interface.

For a clearer quantitative comparison of the compositional evolution observed by EDS, the representative local compositions obtained for the different heat-treatment conditions are summarized in Table 7. The values should be interpreted as local compositional characteristics of the selected microstructural regions rather than as bulk coating compositions. Similar high-temperature redistribution and interdiffusion phenomena have been reported for NiCrAlY/MCrAlY systems subjected to vacuum heat treatment. However, the present results specifically demonstrate that, in the detonation-sprayed NiCrAl/14MoV6-3 system, the 1100 °C condition produces a combination of bulk structural homogenization and pronounced local chemical heterogeneity at the coating–substrate interface.

The quantitative EDS data demonstrate that the compositional evolution of the coating is strongly temperature-dependent and spatially heterogeneous. At 800 °C, the coating remains predominantly Ni-rich, but a localized Al–O-rich region containing 41.30 wt.% Al and 30.26 wt.% O is already detected, indicating that chemical redistribution is locally initiated at this temperature. At 900 °C, the analyzed coating regions are strongly enriched in Ni and Al, with approximately 37–40 wt.% Al and only 3.6–3.9 wt.% Cr, while the coating–substrate compositional boundary remains relatively sharp. At 1000 °C, the analyzed coating regions contain on average 71.97 wt.% Ni, 15.07 wt.% Al, 2.81 wt.% Cr, and 6.34 wt.% Fe, providing direct evidence of increased Fe participation in the near-interface coating region. The strongest local chemical heterogeneity is observed at 1100 °C: Ni-rich regions containing approximately 79 wt.% Ni coexist with a Cr-rich region containing 40.48 wt.% Cr and 5.13 wt.% Fe. These results show that the progression from 800 to 1100 °C is not simply a monotonic homogenization process. Instead, bulk homogenization of the coating is accompanied by increasingly pronounced local elemental redistribution and coating–substrate interdiffusion at the highest treatment temperatures.

### 3.4. Scratch-Test Response and Apparent Void-like Defects

To complement the structural and microstructural characterization, the adhesion-related mechanical response of the coatings was evaluated by progressive-load scratch testing. The third critical load, L_c3_, was selected as the comparative parameter because it corresponds to severe coating damage and loss of interfacial integrity. The obtained values are summarized in Table 8.

The scratch-test results demonstrate a pronounced effect of vacuum heat treatment on the adhesion-related response of the NiCrAl coating. The as-sprayed coating exhibited an L_c3_ value of 38.49 N, whereas heat treatment at 800 and 900 °C increased L_c3_ to approximately 74 and 72 N, respectively. This increase indicates a substantially higher resistance to severe scratch-induced coating damage after moderate-temperature vacuum heat treatment. At 1000 and 1100 °C, L_c3_ decreased to approximately 60 and 59 N, respectively, although these values remained higher than those of the as-sprayed coating. The decrease at the highest temperatures coincides with the increasingly pronounced coating–substrate interdiffusion and the development of localized interfacial defects revealed by SEM/EDS. Therefore, the scratch-test results suggest that the 800–900 °C range provides the highest interfacial stability under the present test conditions, whereas further heating to 1000–1100 °C promotes microstructural homogenization but is accompanied by a reduction in resistance to severe scratch-induced interfacial damage. Considering both the structural evolution and the scratch-test response, 900 °C appears to provide the most balanced condition among the investigated heat-treatment temperatures. At this temperature, substantial microstructural homogenization is achieved while a high critical scratch load (L_c3_ ≈ 72 N) is retained. Treatment at 1000 °C promotes further structural homogenization but is accompanied by a noticeable reduction in L_c3_, whereas treatment at 1100 °C results in the strongest interdiffusion, localized interfacial defect formation, and the lowest scratch resistance among the heat-treated conditions.

To complement the qualitative SEM observations, a semi-quantitative image-based analysis of dark void-like defects was performed for the as-sprayed coating and for all heat-treatment conditions. The calculated apparent area fractions are summarized in Table 9. Because dark BSE contrast may originate not only from true pores but also from local compositional variations, especially in Al- and O-rich regions, the obtained values are interpreted as apparent void-like area fractions rather than absolute porosity.

The image-based analysis reveals a non-monotonic evolution of dark void-like defects with heat-treatment temperature. The apparent area fraction increases from approximately 0.55% in the as-sprayed coating to 1.42% after treatment at 800 °C. However, this increase should be interpreted with caution because the EDS results for the 800 °C condition show that some dark BSE regions correspond to Al–O-rich, chemically distinct areas rather than true voids. At 900 and 1000 °C, the apparent void-like area fraction decreases markedly to approximately 0.19% and 0.23%, respectively, which is consistent with structural homogenization, solid-state sintering, and partial healing of interlamellar defects. After treatment at 1100 °C, the apparent defect area increases again to approximately 0.89%, mainly due to the development of localized dark void-like features near the coating–substrate interface. This result supports the interpretation that bulk densification and interfacial defect formation occur simultaneously at the highest treatment temperature.

Another important feature of the sample after treatment at 1100 °C is the significantly more complex morphology of the upper surface. Unlike the relatively smooth interior of the coating, the outer boundary becomes distinctly wavy and contains individual protruding and dark-contrast regions. The oxygen map shows its predominant accumulation specifically in this surface region. This means that even under vacuum treatment, some interaction between the coating and oxygen occurs. The most sensitive element is Al due to its very high chemical affinity for oxygen. Therefore, at 1100 °C, the surface redistribution of Al becomes particularly pronounced: Al diffuses toward the surface and can react with oxygen to form an Al–O-containing film. The Cr map after treatment at 1100 °C is no longer completely uniform: an increased signal is observed near certain regions of the interface. Such behavior is associated with changes in the thermodynamic stability of the γ, β, and Cr-rich phases [54]. High-temperature interdiffusion of Al and Cr in MCrAlY systems is closely interconnected. Recent studies show that at temperatures of approximately 1080–1100 °C, the diffusion fluxes of Al and Cr become comparable and significantly determine the morphology of the interdiffusion zone.

### 3.5. Implications for Coating Stability and Study Limitations

From the standpoint of high-temperature service, the observed phase and microstructural evolution can have both beneficial and detrimental effects on coating performance. The retention of β-NiAl is potentially beneficial because this phase provides an Al reservoir for the formation and maintenance of protective Al_2_O_3_-rich scales during subsequent high-temperature exposure. Detonation-sprayed NiCrAlY coatings have been reported to exhibit favorable oxidation resistance at approximately 1050 °C owing to the development of a protective alumina-rich oxide scale [50]. However, continued Al consumption and redistribution of the Ni–Al phases during prolonged thermal exposure can progressively reduce the amount of Al available for protective-scale formation. At the same time, the pronounced coating–substrate interdiffusion observed at 1000–1100 °C may adversely affect long-term coating stability. Interdiffusion in MCrAlY-coated systems can produce substantial redistribution of Al and other alloying elements and modify the coating–substrate transition region [54]. The local microvoids observed near the interface at 1100 °C may additionally act as stress-concentration sites and thereby facilitate crack initiation or interfacial damage during repeated thermal cycling. Therefore, the combination of enhanced interdiffusion and microvoid formation observed at 1100 °C is potentially unfavorable for long-term mechanical integrity. Conversely, appropriately selected heat-treatment conditions may improve coating performance by promoting microstructural homogenization and reducing structural defects. Previous studies of NiCrAlY coatings have demonstrated that coating microstructure, porosity, adhesion, and oxidation resistance are closely interrelated [2]. For the present NiCrAl/14MoV6-3 system, the 900–1000 °C range therefore appears structurally more balanced than 1100 °C, because substantial homogenization occurs without the pronounced interfacial heterogeneity and microvoid formation observed at the highest treatment temperature. Nevertheless, adhesion strength, cyclic oxidation, thermal fatigue, and long-term service life were not directly evaluated in the present study; consequently, these performance implications should be regarded as mechanistically supported expectations rather than experimentally demonstrated lifetime improvements. Therefore, the 900–1000 °C range is considered structurally favorable only in the context of the present XRD, SEM, EDS, and roughness results, while confirmation of its technological optimum would require complementary mechanical and high-temperature oxidation testing.

A limitation of the present study is that the absolute chamber pressure was not recorded during vacuum heat treatment. Consequently, although the phase evolution of the oxide-containing constituents is clearly identified by XRD and supported by EDS observations, the relationship between oxide formation and oxygen partial pressure cannot be quantified.

## 4. Conclusions

The conducted study showed that vacuum heat treatment has a significant effect on the structural-phase state, microstructure, and surface morphology of detonation-sprayed NiCrAl coatings. The as-sprayed coating is characterized predominantly by the presence of β-NiAl, pronounced structural non-equilibrium, inherited lamellar morphology, and individual inter-splat defects formed under conditions of high-velocity detonation deposition and rapid particle cooling.

It was established that increasing the vacuum treatment temperature from 800 to 1100 °C leads to a sequential restructuring of the coating. At 800 °C for 4 h, active diffusional redistribution of Ni, Al, and Cr begins, accompanied by the formation of several Ni–Al- and Cr-containing intermetallic phases. At the same time, the lamellar structure is still well preserved, while Al–O-rich regions are detected in certain areas, indicating local interaction of Al with oxygen even under vacuum conditions.

After treatment at 900 °C, more pronounced phase and structural homogenization is observed. The bulk of the coating becomes more homogeneous, the prominence of interlamellar boundaries decreases, and the distribution of Ni and Al becomes more uniform. β-NiAl is retained as one of the main phases; however, the relative content of intermediate Ni–Al compounds changes, indicating a further approach of the system toward a more stable phase state.

At 1000 °C, along with the continuing homogenization of the metallic constituent, two additional processes begin to manifest: the formation of crystalline oxide phases and enhanced interdiffusion with the substrate. NiO and Cr_2_O_3_ reflections appear in the XRD patterns, while SEM/EDS reveals the presence of Fe in the lower part of the coating and increasing complexity of the transition zone. Thus, this condition represents a transition between predominantly intralayer structural stabilization and intensive interaction within the coating–substrate system.

The most significant changes are observed after treatment at 1100 °C for 4 h. The initial lamellar structure of the coating largely disappears, and the bulk of the layer becomes more homogeneous; however, the complexity of the phase composition increases sharply. In addition to Ni–Al intermetallic phases, Al_2_O_3_, Cr_2_O_3_, NiO, and NiAl_2_O_4_ are detected. A chemically and morphologically heterogeneous zone with local microvoids also forms near the interface, which may be associated with unequal interdiffusion fluxes of Fe, Ni, Al, and Cr and possible Kirkendall-type vacancy accumulation.

Combined XRD, SEM, and EDS analysis shows that the phase evolution of the coating is non-monotonic and is governed by the competition between several processes: relaxation of the initial microstrains, crystallite growth, diffusion-driven homogenization, redistribution of β-NiAl and other Ni–Al phases, selective oxidation of Al and Cr, and interdiffusion with the substrate. In the temperature range of 800–900 °C, ordering and homogenization of the metallic Ni–Cr–Al system dominate, whereas at 1000–1100 °C oxidation processes and coating–substrate interactions begin to play an increasingly important role.

Visual analysis of the surface and profilometry results confirm the overall trend of thermal restructuring. Increasing the temperature from 800 to 1000 °C is accompanied by moderate surface smoothing: surface roughness exhibited a non-monotonic dependence on heat-treatment temperature. The Ra values were 6 ± 0.20, 5.3 ± 0.18, 5.3 ± 0.19, and 5.9 ± 0.21 μm at 800, 900, 1000, and 1100 °C, respectively. The minimum roughness was therefore obtained at 900 °C, whereas further heating to 1100 °C resulted in a moderate increase in surface roughness. This reduction is associated with enhanced surface and interlamellar diffusion, solid-state sintering, and relaxation of structural defects. At the same time, the temperature-induced change in surface color reflects changes in the composition and morphology of thin surface oxide and intermetallic layers, which is consistent with the XRD results.

Considering both the structural evolution and the scratch-test response, 900 °C appears to provide the most balanced condition among the investigated heat-treatment temperatures. At this temperature, substantial microstructural homogenization is achieved while a high critical scratch load (L_c3_ ≈ 72 N) is retained. Treatment at 1000 °C promotes further structural homogenization but is accompanied by a noticeable reduction in L_c3_, whereas treatment at 1100 °C results in the strongest interdiffusion, localized interfacial defect formation, and the lowest scratch resistance among the heat-treated conditions.

## Figures and Tables

**Table 1:** Nominal chemical composition, representative mechanical properties, and rationale for the selection of 14MoV6-3 steel as the substrate material.

Parameter	Value/Range	Relevance to the Present Study
C, wt.%	0.10–0.15	Provides strength while maintaining weldability and thermal stability
Si, wt.%	0.15–0.35	Contributes to deoxidation and solid-solution strengthening
Mn, wt.%	0.40–0.70	Improves strength and toughness
Cr, wt.%	0.30–0.60	Contributes to high-temperature strength and oxidation resistance
Mo, wt.%	0.50–0.70	Improves creep and high-temperature strength
V, wt.%	0.22–0.28	Promotes precipitation strengthening and thermal stability
Ni, wt.%	≤0.30	Residual/alloying constituent
Cu, wt.%	≤0.30	Residual constituent
P, wt.%	≤0.025	Controlled impurity
S, wt.%	≤0.010	Controlled impurity
Fe	Balance	Matrix constituent
Yield strength, R_p0.2_	≥320 MPa *	Provides sufficient mechanical support for the coating
Tensile strength, R_m_	460–610 MPa *	Characteristic strength range of the substrate
Elongation, A	≥20% *	Indicates adequate substrate ductility
High-temperature capability	Used for elevated-temperature pressure-service applications	Makes the steel relevant as a substrate for evaluating heat-resistant NiCrAl coatings

* Values representative of 14MoV6-3 according to ISO 4955 for small section thicknesses; they were not measured independently in the present work.

**Table 2:** Technological parameters of the detonation spraying process for producing NiCrAl coatings.

Parameter	Value	Unit
O_2_/C_2_H_2_ ratio	1.856	dimensionless
Barrel filling with explosive gas	50	%
Spraying distance	150	mm
Number of detonation shots	20	shots
Barrel inner diameter	20	mm
Barrel length	~800	mm
Carrier gas	N_2_	-
As-sprayed coating thickness	~85–90	μm

**Table 3:** The technological parameters of vacuum heat treatment of detonation-sprayed NiCrAl coatings.

Treatment Temperature°C	Treatment Time, h	Heating Rate, °C/min	Cooling Rate, °C/min
800	4	15.1	4.2
900	4	15	3.75
1000	4	14.9	3.9
1100	4	14.8	4

**Table 4:** Reference diffraction patterns used for phase identification of the NiCrAl coatings.

Phase	Structural Designation	Reference Code
β-NiAl	AlNi	96-900-8803
Al_4_Ni_3_	Al_4_Ni_3_	00-046-1037
AlNi_3_	Ni_3_Al/AlNi_3_	00-050-1265
Al_3_Ni_2_	Al_3_Ni_2_	00-014-0648
Cr_2_Ni_3_	Cr_2_Ni_3_	03-065-6291
AlCr_2_	AlCr_2_	03-065-6360
Cr_2_O_3_	chromium(III) oxide	00-038-1479
NiO	nickel oxide	00-047-1049
α-Al_2_O_3_	aluminum oxide	00-011-0661
NiAl_2_O_4_	nickel aluminate spinel	03-065-3102

**Table 5:** Semi-quantitative normalized relative diffraction contributions of selected reflections for the NiCrAl coating before and after vacuum heat treatment.

Condition	β-NiAl, %	Al_4_Ni_3_, %	AlNi_3_, %	Al_3_Ni_2_, %	Oxides/Spinel, %
As-sprayed	64.0	18.3	11.6	2.2	4.0
800 °C	81.7	11.0	0.1	5.8	1.4
900 °C	88.1	0.6	5.2	4.5	1.6
1000 °C	85.5	0.1	9.2	1.6	3.7
1100 °C	42.1	29.8	16.4	3.2	8.5

**Table 6:** Surface roughness of the NiCrAl coatings after vacuum heat treatment at different temperatures.

Heat-Treatment Temperature ◦C	Ra, μm	Number of Measurements
800	6 ± 0.20	5
900	5.3 ± 0.18	5
1000	5.3 ± 0.19	5
1100	5.9 ± 0.21	5

**Table 7:** Representative local EDS compositions of the detonation-sprayed NiCrAl coating after vacuum heat treatment.

Condition	Analyzed Region	Ni, wt.%	Al, wt.%	Cr, wt.%	Fe, wt.%	O, wt.%
800 °C	Ni-rich matrix, spectra 1, 2, 4, 5	74–81	11.6–18.0	3–9	—	—
800 °C	Local Al–O-rich region, spectrum 3	23.72	41.30	minor	—	30.26
900 °C	Spectrum 1	58.90	37.20	3.90	—	—
900 °C	Spectrum 2	56.93	39.50	3.56	—	—
1000 °C	Spectra 1–5, range	69.46–76.30	12.07–17.15	2.36–3.07	3.21–8.36	—
1000 °C	Mean of selected points	71.97	15.07	2.81	6.34	—
1100 °C	Spectrum 1, Ni-rich region	79.37	12.82	4.38	—	—
1100 °C	Spectrum 5, Ni-rich region	79.14	13.32	4.05	—	—
1100 °C	Spectrum 3, Cr-rich region	16.92	40.48		5.13	—

Note: The EDS values correspond to representative local regions identified in the SEM/BSE images. They are not statistically averaged bulk compositions. A dash indicates that the corresponding element was not quantitatively reported for that spectrum.

**Table 8:** Critical scratch load L_c3_ of the NiCrAl coatings before and after vacuum heat treatment.

Condition	L_c3_, N
As-sprayed	38.49
800 °C	74
900 °C	72
1000 °C	60
1100 °C	59

**Table 9:** Image-based apparent area fraction of dark void-like defects in the NiCrAl coatings before and after vacuum heat treatment.

Condition	Apparent Void-like Area Fraction, %
As-sprayed	0.55
800 °C	1.42
900 °C	0.19
1000 °C	0.23
1100 °C	0.89

## Data Availability

The original contributions presented in this study are included in the article. Further inquiries can be directed to the corresponding author.
